# PEDOT: PSS‐Enabled CNT and Bi_0.5_Sb_1.5_Te_3_ Interface Microengineering for Integrated Thermoelectric Energy Harvesting and Corrosion Protection in Cementitious Composite

**DOI:** 10.1002/advs.202508424

**Published:** 2025-08-19

**Authors:** Chen Zhang, Yingwu Zhou, Feng Xing, Menghuan Guo, Jiawei Wu, Junfeng Wang, Yinghui Wu

**Affiliations:** ^1^ Guangdong Provincial Key Laboratory of Durability for Marine Civil Engineering Shenzhen University Shenzhen 518060 P. R. China; ^2^ School of Mechanics and Construction Engineering Jinan University Guangzhou 51063 P. R. China

**Keywords:** CNT cementitious composites, PEDOT: PSS, Bi₀._5_Sb₁._5_Te_3_, self‐powered cathodic protection, thermoelectric properties

## Abstract

Thermoelectric cement‐based composites integrate thermoelectric effects with structural capabilities, presenting an effective solution for harvesting environmental heat in self‐powered cathodic protection. While the prospects are promising, their performance has been constrained by the compatibility between functional fillers and cementitious materials. This study demonstrates that PEDOT: PSS(PP) significantly improves the dispersion of multi‐walled carbon nanotube (CNT) and Bi_0.5_Sb_1.5_Te_3_ (BST) in cementitious materials. The optimized composite(0.2 wt.% CNT, 1.0 vol% PP, and 1.0 wt.% BST) exhibits a 28.4% increase in conductivity and a 15.9% reduction in thermal conductivity compared to the control. Additionally, it achieves an impressive Seebeck coefficient of 450 µV K^−1^. Importantly, the composite maintains superior compressive strength (> 40 MPa) and chloride penetration resistance (< 7 × 10^−12^ m^2^ s^−1^), with over 80% property retention after 60 days under extreme temperatures of −20 or 70 °C. A thermoelectric generator (TEG) is assembled by connecting 30 specimens in series to form a 10 × 10 cm^2^ device. The TEG exhibits less than 8% voltage decay during 20 h of continuous operation and successfully powered an LED. The TEG also substantially mitigates steel corrosion in self‐powered cathodic protection, reducing corrosion current density and corrosion rate by more than 47%.

## Graphical abstract

In this study, PP and BST are used to synergistically enhance the dispersibility and thermoelectric performance of CNT cementitious materials. Further Author Manuscript research shows that the composite material maintains >80% compressive strength and chloride resistance performance at −20 or 70°C. A TEG assembled from 30 series‐connected specimens successfully powers a commercial LED and achieves a power supply for steel cathodic protection.

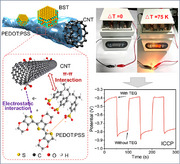

## 1 Introduction

Reinforced concrete structures are frequently subjected to structural damage due to environmental corrosion, which significantly impacts their long‐term durability.^[^ ^1^, ^2^ ^]^ Conventional impressed current cathodic protection systems, which rely on external power sources, are associated with high maintenance costs and limited applicability.^[^ ^3^ ^]^ In the context of carbon neutrality, there is an increasing demand for self‐powered solutions within the construction industry. Thermoelectric materials, capable of converting ambient waste heat into electrical energy, offer advantages such as continuous operation and minimal maintenance, making them suitable for decentralized heat recovery in buildings.^[^ ^4^, ^5^ ^]^ Incorporating the thermoelectric effect into cementitious materials not only facilitates self‐powered structures but also provides continuous cathodic protection for steel reinforcement, thereby enhancing the sustainability and durability of the building. Consequently, the development of thermoelectric cementitious composites for the cathodic protection of reinforced concrete is of paramount importance.

The enhancement of thermoelectric performance in cementitious composites has been actively pursued through the incorporation of functional fillers, including carbon‐based additives, metal compounds/oxides, and nanofibers.^[^ ^6^, ^7^, ^8^ ^]^ Carbon‐based additives, such as carbon fibers, graphene, and carbon nanotubes (CNT), offer exceptional electrical conductivity (*σ*) and enhance mechanical performance. However, these materials typically exhibit p‐type semiconducting behavior in the cement matrix, resulting in a limited Seebeck coefficient (*S* = 13.7–34 µV K^−1^), which restricts their thermoelectric efficiency.^[^ ^9^, ^10^, ^11^, ^12^ ^]^ Transition metal oxides, such as MnO_2_, ZnO, and Fe_2_O_3_, show improved *S* but possess inherently low conductivity, requiring high filler substitution ratios, which may compromise the mechanical properties of the composite.^[^ ^13^, ^14^, ^15^ ^]^ Similarly, industrial wastes, such as silica fume and fly ash, demonstrate moderate thermoelectric properties as functional fillers.^[^ ^16^, ^17^, ^18^ ^]^ Wen and Chung^[^ ^19^ ^]^ reported a maximum *S* of 31 µV K^−1^ for steel fiber‐reinforced cement pastes. In contrast, Bi_2_Te_3_ exhibits both superior σ and S simultaneously. Liu et al.^[^ ^20^ ^]^ incorporated 0.6 wt. % Bi_2_Te_3_ into cementitious materials, achieving a more than 10 times increase in *S*. Moreover, Bi_2_Te_3_ doped as a coating within the cement matrix resulted in a higher *S*. The use of composite multiphase functional fillers has shown promise in providing balanced thermoelectric enhancement. Vareli et al.^[^ ^21^ ^]^ combined CNT with nano carbon black to achieve a significant enhancement of *S* without sacrificing *σ*. Ji et al.^[^ ^22^ ^]^ demonstrated the feasibility of cathodic protection using PANI/MnO_2_‐cement composites, their practical application is constrained by brittleness at high filler substitution ratios. The Wei group's approach, combining metal oxides (Ca_3_Co_4_O₉, CuO, TiO_2_) with carbon materials, improved thermoelectric properties but introduced interfacial incompatibility.^[^ ^23^, ^24^, ^25^ ^]^ Additionally, while specialized treatments such as acid or heat treatment can enhance the *S*, these methods often induce interfacial contact resistance or intensify carrier scattering, potentially negating conductivity improvements.^[^ ^26^, ^27^ ^]^ Despite these advancements, the challenges associated with the dispersion of functional fillers in alkaline cementitious environments remain a significant barrier to practical implementation.

Conductive polymers, such as PEDOT: PSS (PP), PPy, and PANI, have shown considerable potential in a wide range of applications due to their unique interfacial modification properties, presenting a promising solution for the enhancement of cementitious composites.^[^ ^28^, ^29^ ^]^ Specifically, PP, as a P‐type material, exhibits a conductivity range of 0.1–10 S cm^−1^ and a *S* of ≈15–18 µV K^−1^, while maintaining stable electrical conductivity even in highly alkaline environments.^[^ ^30^, ^31^ ^]^ Additionally, Liu et al.^[^ ^32^ ^]^ reported CNT/PP treated with NaOH aqueous solution achieved a low thermal conductivity (*κ*) of 0.4–0.6 W m^−1^ K^−1^.While the thermoelectric properties of PP have been extensively studied, whether its advantages, such as high electrical conductivity and a high *S*, are maintained in cementitious composites (pH > 12.5) requires further systematic investigation.

In this work, we present the design of a thermoelectric cementitious material (CNT/PEDOT: PSS/Bi₀._5_Sb₁._5_Te_3_(BST)) for environmental waste heat energy harvesting. A PP aqueous solution was utilized as a dispersant to prevent CNT agglomeration through its hydrophilicity and spatial site‐blocking effect. BST nanoparticles were incorporated to construct a CNT/PP/BST hybrid conductive network. This ternary enables simultaneous optimization of *σ*, *S*, and *κ*. The Sb‐doped BST phase demonstrates superior alkaline stability compared to Bi_2_Te_3_.^[^ ^33^ ^]^ The cement‐based composites retain over 80% of their performance after 60 days of exposure to extreme temperature conditions of ‐20 and 70 °C. A thermoelectric generator (TEG) with 10 × 10 cm^2^was assembled by connecting 30 thermoelectric legs in series. The TEG successfully powered a commercial LED and was also applied for the cathodic protection of steel bars, demonstrating a self‐powered function.

## 2 Results and Discussion

### 2.1 Dispersibility of Carbon Nanotubes (CNT)/PEDOT: PSS (PP)/Bi_0.5_Sb_1.5_Te_3_(BST) Cement‐Based Composites

**Figure 1:**
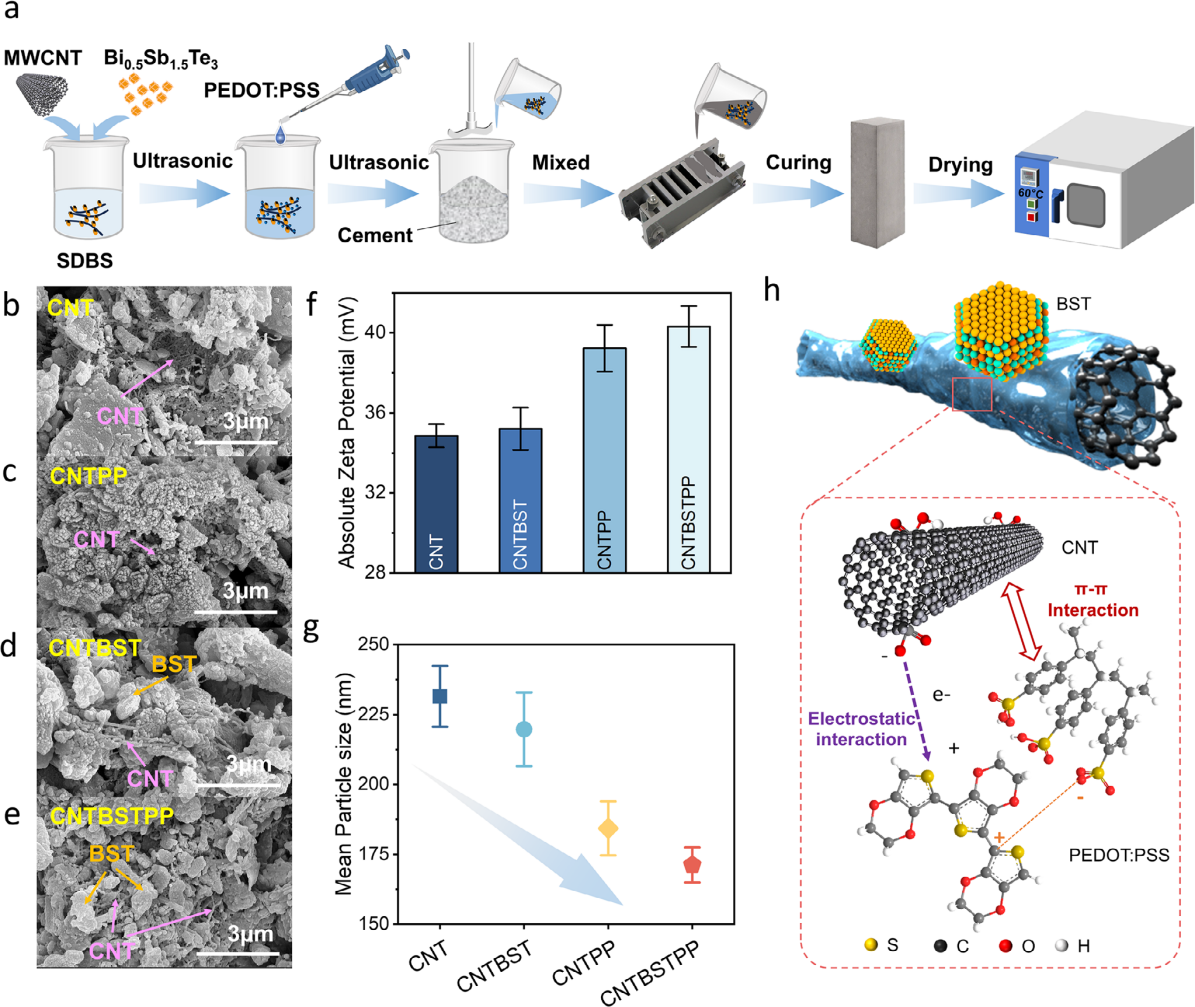
a) CNT/PEDOT: PSS/Bi_0.5_Sb_1.5_Te_3_ cement‐based material preparation process. SEM surface image of b) CNT, c) CNTPP, d) CNTBST, e) CNTBSTPP. f) Zeta potential of CNT/PEDOT: PSS/Bi0.5Sb1.5Te3 cement‐based composites. g) Mean particle size of DLS. h) The mechanism diagram of PEDOT: PSS promoting CNT dispersion.

**Figure** **1**a schematically illustrates the fabrication process of the CNT/PEDOT: PSS(PP)/BST cementitious composite, with details provided in the Supporting Information. Figure 1b–e reveals that the CNT surfaces acquire a coarser texture following PP modification, confirming successful adhesion of PP to the CNT surfaces. In the ternary CNT/PP/BST composite system, individual CNTs and small nanotube bundles are uniformly embedded within the cement matrix. At the same time, BST particles remain homogeneously dispersed around the CNT networks without macroscopic aggregation. This optimized microstructure facilitates the establishment of continuous CNT/PP/BST conductive pathways, critical for enhancing electrical transport. Energy‐dispersive X‐ray spectroscopy (EDS) mapping (Figure S1, Supporting Information) further confirms spatial correlation of *S* (from PP) and Bi (from BST) elements within the carbon‐rich regions, supporting interfacial interactions between components. Comparison with pristine BST particles (Figure S2, Supporting Information) confirms morphological integrity and retention of BST throughout composite processing. Colloidal stability assessments (Figure S3, Supporting Information) reveal that pristine CNT in sodium dodecylbenzene sulfonate (SDBS) solutions aggregate after 24‐h sedimentation, while the CNT/PP system maintains stability under identical conditions, highlighting the PP's role in enhancing CNT dispersion and interfacial compatibility in aqueous media. Zeta potential and dynamic light scattering (DLS) analyses further demonstrate enhanced electrostatic stabilization in the ternary composite. Figure 1f,g shows that the CNT/PP/BST system exhibits a 5.46 mV increase in zeta potential and a 40.3 nm reduction in average diameter compared to unmodified CNT. A smaller particle size can prevent the interruption or discontinuity of the conductive path that may result from agglomeration, while a higher Zeta potential indicates strong interfacial contact between filler particles.^[^ ^11^ ^]^ Consequently, the transfer and transport of electrons are promoted, thereby enhancing the composite's conductivity and Seebeck coefficient. The superior dispersion of both CNT and BST within the PP matrix arises from synergistic stabilization mechanisms: oxygen‐containing groups on the CNT surface block certain spatial sites when interacting with PP,^[^ ^34^, ^35^ ^]^ while *π–π* interactions reduce functional fillers aggregation and promote uniform distribution (Figure 1h). This ultimately contributes to the formation of a well‐bridged structure within the cement matrix.

### 2.2 Material Characterization of CNT/PP/BST Cement‐Based Composites

**Figure 2:**
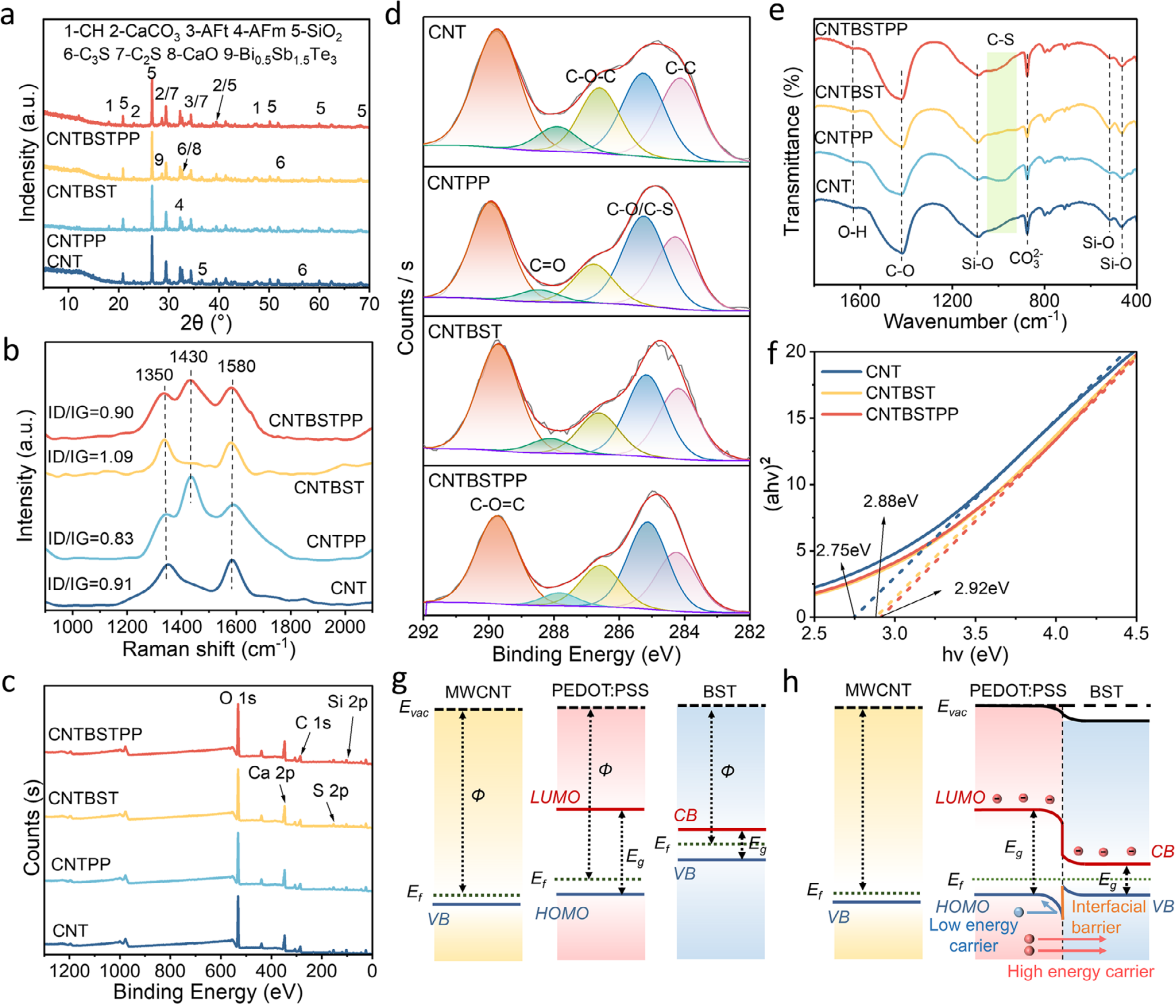
Microscopic characterization of CNT/PP/BST cement‐based composites. a) XRD patterns of CNT/PP/BST cement‐based composites. b) Raman spectra of CNT/PP/BST cement‐based composites. c) XPS spectra of CNT/PP/BST cement‐based composites. d) C 1s peak of XPS spectra. e) FTIR spectra of CNT/PP/BST cement‐based composites. f) Tauc plots. Energy band diagram of the ternary system before g) and after contact h).

The structural evolution and interfacial interactions within the CNT/PP/BST composite system were investigated. **Figure** **2**a confirms phase integrity preservation in the CNT/PP/BST composite, with the characteristic BST peak at 27.7° (Figure S4, Supporting Information) and cement hydration phases (C_3_S, CaCO_3_, AFm) remaining unperturbed. This suggests that the PP/BST modification does not interfere with the cement hydration reaction. Figure 2b further elucidates interfacial coupling mechanisms. The characteristic carbon D‐band (1350 cm^−1^) and G‐band (1580 cm^−1^) persist in all composites, while the PP vibrational mode at 1430 cm^−1^ confirms polymer retention.^[^ ^35^ ^]^ The PP addition exhibits a decrease in the G peak intensity and ID/IG ratio, suggesting effective wrapping of CNT and BST. Similar observations were reported in the PEDOT: PSS / graphene composite system.^[^ ^36^, ^37^ ^]^ Furthermore, the G‐band in the ternary composite was blue‐shifted from 1580 to 1586 cm^−1^ (Figure S5, Supporting Information), indicating enhanced charge transfer.^[^ ^38^ ^]^ X‐ray photoelectron spectroscopy (XPS) analysis of chemical bonding states is provided in Figure 2c,d and Table S1 (Supporting Information). The C 1s spectra exhibit a consistent CaCO_3_ signature at 289.5 eV (C‐O = C)^[^ ^39^ ^]^ across all samples, confirming unaltered cement hydration pathways. The dominant C‐C peak at 284.3 eV originates from sp^2^‐hybridized CNT carbons and conjugated PP backbones, while the C‐S bonding signature at 285.6 eV confirms retained PSS sulfonic acid groups.^[^ ^37^, ^40^ ^]^ Furthermore, the O 1s spectra show the C = O bonds (≈533 eV) and C‐O bonds (≈531 eV), which could be attributed to the oxygen‐containing groups introduced by CNT surface acidification (Figure S6 and Table S2, Supporting Information).^[^ ^40^ ^]^ These results indicate that PP and BST critically preserve the oxygen‐containing groups on the CNT surface, thereby enhancing the stability of the CNT‐cement matrix interface. Fourier‐transform infrared spectroscopy (FTIR, Figure 2e) reinforces this interfacial stability, with the preserved C‐S vibrational mode (973 cm^−1^, the characteristic peak of PP)^[^ ^34^ ^]^ confirming the structural integrity of PP.

The UV–vis absorption and Tauc plots curves of the cementitious composites (Figure 2f; Figure S7, Supporting Information) show an increase in the bandgap with the introduction of PP and BST. Kelvin probe force microscope (KPFM) results (Figure S8, Supporting Information) show that the average contact potential difference of CNT and CNTBSTPP are 341and ‐401mV, respectively, which also indicates that the addition of BST and PP increases the work function. Combined with Figure 2g,h, these results demonstrate that the three composites form an excellent energy level arrangement structure. According to previous reports,^[^ ^41^, ^42^ ^]^ the HOMO energy levels of PEDOT: PSS are well matched with the Fermi energy levels of CNT and the valence band of BST. Under varying thermal gradients, the thermal equilibration of the Fermi levels upon contact results in band bending, which in turn creates an energy barrier at the interface. This band bending induces a potential barrier that reflects lower‐energy carriers while allowing higher‐energy carriers to contribute to the generation of the Seebeck coefficient (Figure S9, Supporting Information). Cyclic voltammetry (CV) results indicate that CNT/PP/BST composite exhibits enhanced electrochemical reactivity and stronger double‐layer capacitance characteristics compared to controls (Figures S10 and S11, Supporting Information). This phenomenon implies the improved interfacial charge transfer capability imparted by PP and BST modification. This optimized energy level arrangement effectively reduces the interfacial charge transport barrier, providing efficient transport of hot carriers.

### 2.3 Thermoelectric and Electrothermal Properties of CNT/PP/BST Cement‐Based Composites

**Figure 3:**
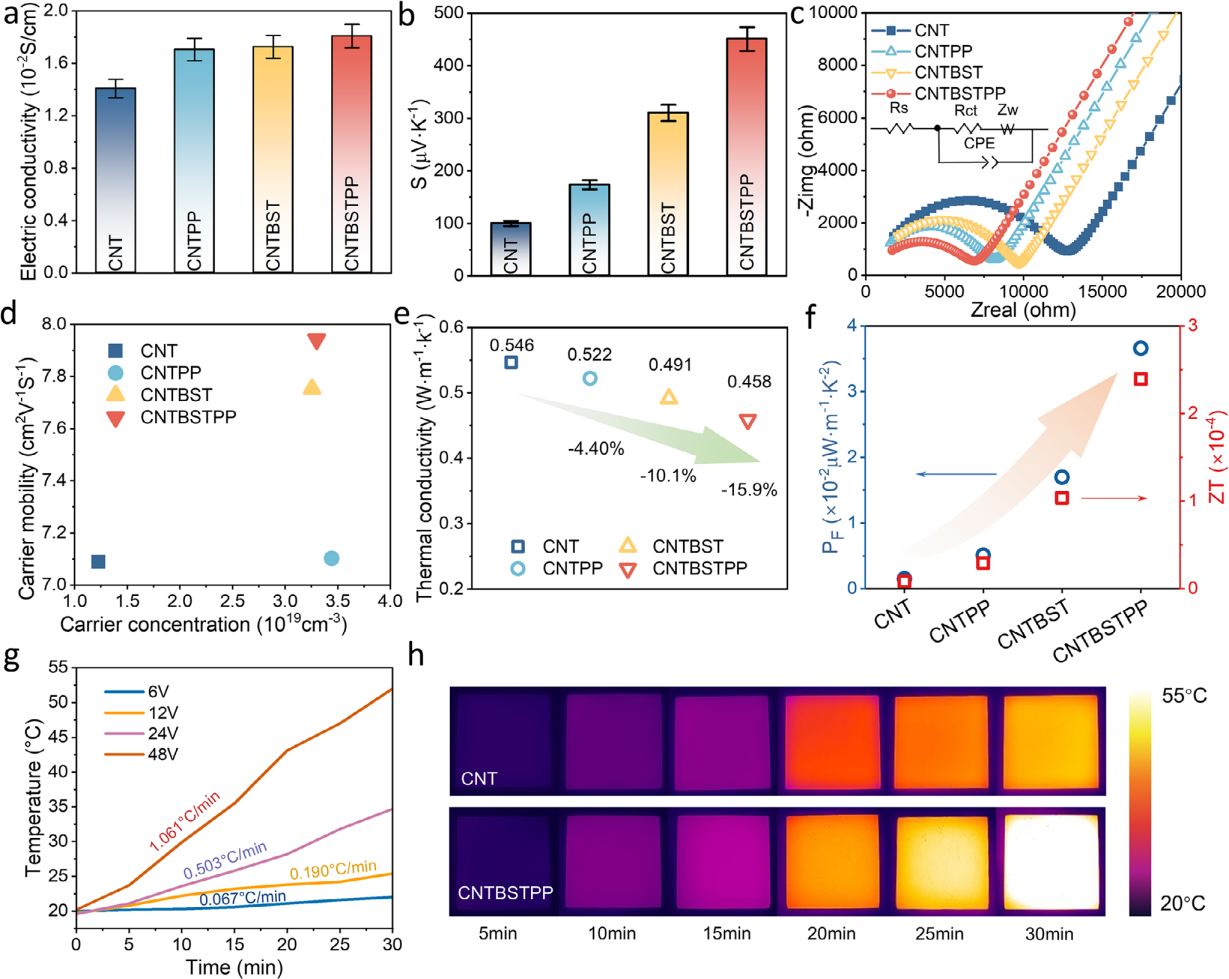
Thermoelectric property of CNT/PP/BST cement‐based composites: a) Electrical conductivity *σ*. b) Seebeck coefficient *S*. c) Nyquist diagram of EIS results. d) Carrier concentration 𝜂_H_ and carrier mobility *µ*_H_. e) Thermal conductivity *κ*. f) Power factor PF and Figure of merit ZT., and electrothermal property: g) Surface temperature of CNTBSTPP at different input voltages and different times. h) Infrared thermal imaging of CNT and CNTBSTPP at different times at 48V.

The thermoelectric properties of the cement composites are shown in **Figure** **3**. Incorporation of PP and BST progressively enhances both the electrical conductivity (*σ*) and Seebeck coefficient (*S*), with the ternary CNT/PP/BST composite exhibiting the highest performance. The optimized formulation ratio of CNTBSTPP has been determined through orthogonal experimental design, which balances *σ*, *S*, and compressive strength (Tables S3–S5, Supporting Information). The *σ* of CNTBSTPP (1.81 × 10^−2^ S cm^−1^) is significantly enhanced compared to the single component system (Figures S12–S14, Supporting Information) and exceeds binary systems (CNTPP: 1.70 × 10^−2^ S cm^−1^, CNTBST: 1.72 × 10^−2^ S cm^−1^) by 5.23%‐6.47% (Figure 3a). The chemical activity and electrical stability of PP were confirmed by FTIR peaks (Figure S15, Supporting Information), Tauc plots (Figure S16, Supporting Information), and *σ* after 60‐day curing (Figure S17, Supporting Information). The FTIR spectrum showed C‐S peaks at 980 cm^−1^ and CNTBSTPP's bandgap of Tauc plots, indicating stability in cement‐based materials. Additionally, CNTBSTPP retained over 95.4% of its 7‐day *σ* after 60‐day curing. Similarly, the *S* increases from 99 µV K^−1^ for pristine CNT to 171 µV K^−1^ for CNTPP and reaches 450 µV K^−1^ for CNTBSTPP at room temperature (Figure 3b). Moreover, by carrying out the electrochemical impedance spectroscopy (EIS) test, the results of interfacial charge dynamic analysis further confirm the above‐mentioned phenomenon. The reduced semicircle diameter in the high‐frequency region (Figure 3c) and lower charge transfer resistance (Table S6, Supporting Information) indicate that PP and BST decrease interfacial resistance, thereby improving electron transfer. To investigate the enhancement of PP and BST, Hall effect experiments were used to test the room‐temperature carrier concentration (η_*H*_) and mobility (µ_*H*_)of these samples (Figure 3d). The enhancement of η_*H*_ and µ_*H*_is attributed to PP's high intrinsic carrier mobility and the formation of an energy barrier at the PP / BST interface, which is favorable for the transport of hot carriers.^[^ ^43^, ^44^ ^]^

The total thermal conductivity (𝜅_tot_) and electronic thermal conductivity (𝜅_e_, calculated by Equation S2, Supporting Information) of the samples are given in Figure 3e and Figure S18 (Supporting Information). While the incorporation of PP and BST elevates 𝜅_e_, the ternary CNT/PP/BST system achieves a net 15.9% reduction in 𝜅_tot_ compared to pristine CNT composites (Figure 3e). This reduction is primarily due to the relatively small contribution of *κ*_e._ The formation of the PP/BST interface reduces the average free path of phonons, which promotes the effective phonon scattering, with the total thermal conductivity*k_tot_* = *k_L_* + *k_e_* remaining lower. Additionally, thermodynamic analysis reveals an enhancement in specific heat capacity (Cp) in the ternary system, improving heat flux retardation and thermal energy storage capabilities (Figure S19, Supporting Information). Benefiting from the optimization of *σ*, *S*, and *𝜅*, significant improvements in power factor (PF) and dimensionless Figure of merit (ZT) are achieved (Figure 3f). PF escalates from 0.015 × 10^−2^ to 0.366 × 10^−2^ µW m^−1^ K^−2^, while ZT surges from 8.28 × 10−⁶ to 2.39 × 10−⁴. To characterize the relationship between dispersion and thermoelectric performance, the zeta potential and DLS particle size were linearly fitted with the thermoelectric performance parameters (Figure S20, Supporting Information). The results demonstrate a strong correlation (R^2^ > 0.9), indicating that the thermoelectric performance is remarkably enhanced by promoting the dispersibility of CNT. Table S7 and Figure S21 (Supporting Information) present the thermoelectric performance and ZT values of current multi‐walled CNT cementitious materials, demonstrating good performance.

Figure 3g demonstrates voltage‐responsive thermal modulation of CNTBSTPP, exhibiting a linear temperature rise gradient from 0.067 °C (6 V) to 1.061 °C min^−1^ at 48 V. Figure 3h shows that CNTBSTPP has a higher temperature rise efficiency, with IR thermography revealing no local hot spots. This phenomenon is mainly attributed to the optimized conductive network in the CNT/PP/BST ternary system, which significantly reduces interfacial contact resistance. Figure S22 (Supporting Information) further validates thermal homogeneity, showing that the surface temperature distribution error (ε_*T*_) of CNTBSTPP is consistently lower than that of CNT throughout the testing process. This phenomenon confirms the improved temperature uniformity.

### 2.4 Mechanical and Chloride Penetration Resistance of CNT/PP/BST Cement‐Based Composites

**Figure 4:**
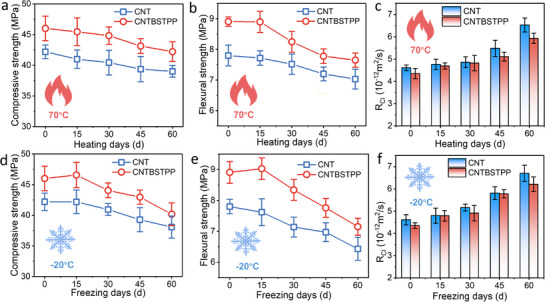
The mechanical properties and resistance to chloride penetration of CNT/PP/BST cement‐based composites in high or low temperature environments. a–c) are the compressive strength, flexural strength, and chloride penetration resistance before and after high temperature (70 °C), respectively. d–f) are the compressive strength, flexural strength, and chloride penetration resistance before and after low temperature (‐20 °C), respectively.

The mechanical properties critically influence the durability and stability of thermoelectric cementitious composites. **Figure** **4** shows the mechanical and chloride penetration resistance properties of CNT/PP/BST cement composites after exposure to high or low temperatures. The improvement of mechanical properties is attributed to the enhanced dispersion of PP‐assisted nanoparticles and the physical filling effect of CNT.^[^ ^45^ ^]^ Under ambient conditions, CNTBSTPP achieves significant mechanical property improvements over CNT. Compressive strength increases by 9.03%, flexural strength by 14.23%, and the chloride diffusion coefficient (R_Cl_) decreases by 5.64%. After 60 days of high‐temperature exposure, CNTBSTPP maintains superior performance, with compressive and tensile strengths of 42.22 and 7.65 MPa, respectively. These values represent increases of 8.23% and 8.82% compared to the CNT composite after similar exposure. This enhanced mechanical stability was accompanied by a 9.19% reduction in R_Cl_ relative to CNT (Figure 4a–c). This phenomenon arises from high‐temperature‐induced water evaporation within the cement matrix, generating microcracks and harmful pores that compromise matrix integrity. The structural defects create percolation pathways for chloride penetration while decreasing compressive and tensile strength. Nevertheless, the elevated mechanical strength and elastic modulus of the BST phase provide structural reinforcement at high temperature. The bridging effect between the CNT/PP/BST network and the cement matrix contributes to sustained reinforcement and toughening.

Cryogenic durability assessments reveal that the CNT/PP/BST composite maintains exceptional mechanical performance. As demonstrated in Figure 4d–f, the ternary system maintains 80.3% compressive strength retention and 81.1% tensile strength preservation after 60‐day exposure at ‐20 °C, demonstrating 1.08 times and 1.11 times improvements over CNT, respectively. Concurrently, CNTBSTPP's R_Cl_ stabilizes at 92% of CNT's. Cryogenic conditions induce thermodynamically driven ice crystallization within the cementitious matrix, generating localized frost heave pressures. This phenomenon leads to the initiation and propagation of internal microcracks, resulting in mechanical strength reduction and chloride penetration acceleration. Remarkably, the CNT/PP/BST composite maintains exceptionally low‐temperature ductility and toughness, attributable to optimized dispersion and hierarchical bridging architecture. Meanwhile, BST exhibits excellent crystalline structural stability at low temperatures,^[^ ^33^ ^]^ which reduces frost heave stresses and suppresses brittle fracture in the cement matrix. FTIR and Raman spectroscopy confirm chemical stability after 60‐day thermal aging and cryogenic exposure (Figure S23, Supporting Information). Results reveal that characteristic peaks persist at 980 cm^−1^ (C‐S bond of PP) in FTIR spectra and 1430 cm^−1^ in Raman spectra. These results collectively demonstrate the retention of PP's structural integrity. In conclusion, the construction of the CNTPPBST network enhances thermal stability, while interfacial toughening contributes to maintaining the mechanical performance and chloride penetration resistance of the matrix at extreme temperatures.

### 2.5 Output Performance of the Thermoelectric Generator (TEG)

**Figure 5:**
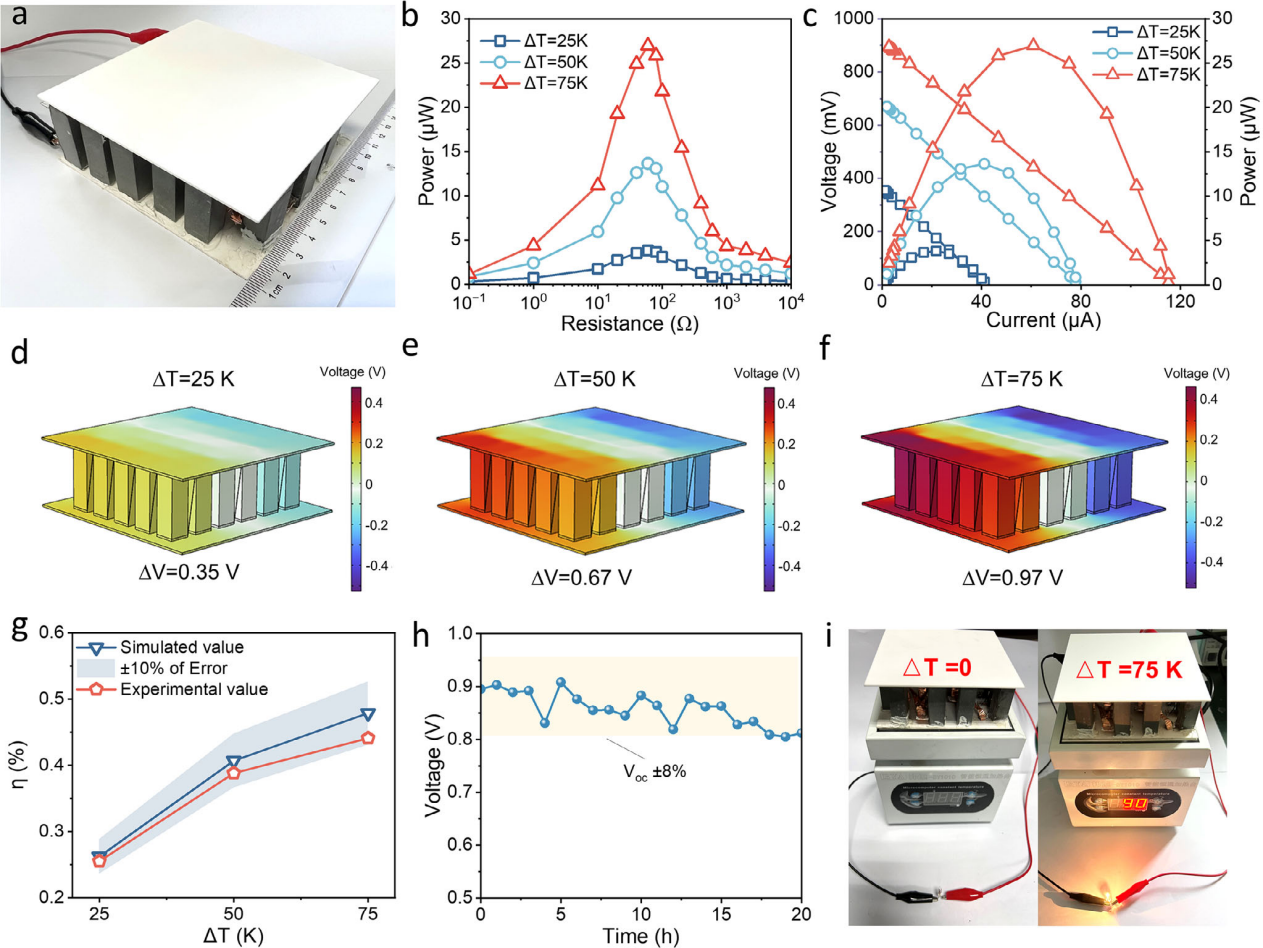
a) Optical image of the fabricated TEG. b) Relationship between output power P and external resistance R_L_. c) Relationship between output power P, output voltage V_oc,_ and current I. d–f) multi‐physics simulation of TEG conversion efficiency at ΔT = 25K, 50K, 75K. g) Error between simulated potential difference and measured value. h) The output voltage of TEG after continuous operation for 20 h. i) TEG supplies power to an LED at ΔT = 0 and 75K.

To evaluate practical thermoelectric conversion capabilities and validate waste heat recovery potential, a modular thermoelectric generator (TEG) measuring 10 cm × 10 cm^2^ was constructed by serial integration of 30 cement‐based elements (10 mm × 10 mm × 30 mm), as depicted in **Figures** **5**a and S24 (Supporting Information). Output Power was tested at temperature differences (ΔT) of 25 to 75 K, with an external load resistor (R_L_). Output power‐load resistance and output power/output voltage‐current curves (Figure 5b,c) were performed for the TEG at ΔT = 25, 50, and 75 K. The output power of the TEG device was calculated by Equation S4 (Supporting Information). As shown in Figure 5b, the output power reaches its maximum at a specific ΔT when the external resistive load is optimally matched with the internal resistance (R_in_). Additionally, the open‐circuit voltage (V_oc_) decreases with increasing current (I). As ΔT increases from 25 to 75 K, V_oc_ rises from 0.33 to 0.89 V, while V_oc_ increases with higher external load (Figure S25, Supporting Information). Furthermore, the performance of the TEG under varying temperature gradients was simulated using COMSOL software (Figure S26, Supporting Information). Figure 5d–f show the potential difference of 0.35, 0.67, and 0.97 V for the simulations at ΔT = 25, 50, and 75 K, respectively, which are also within 8% error from the measured values (Figure S27, Supporting Information). Based on this model, the energy conversion efficiency (*η*), was calculated for the TEG at different ΔT values:^[^ ^46^ ^]^

(1) $$\eta =\frac{P_{out}}{P_{out}+Q_{out}}=\frac{P_{out}}{P_{out}+\kappa \cdot \frac{T_{1}-T_{2}}{H}\cdot \left(L\times W\right)}$$

where *κ* is the thermal conductivity of the heat flux meter, W m^−1^K^−1^. *L* and *W* are the two sides of the heat flow meter section, mm. *H* is the vertical distance between *T*_1_ and *T*_2_ temperature measurement points on the heat flow meter, mm.

The error between calculated and experimental values is shown in Figure 5g. The maximum *η* is 0.44%, while the error from the measured value is 7.74% at ΔT = 75 K. The *η* prediction errors remain within ±10%, indicating the accuracy of the model. Continuous operation testing (Figure 5h) demonstrates excellent stability with <8% voltage decay after 20 h at ΔT = 75 K. The series‐connected TEG successfully powered a commercial light, as shown in Figure 5i.

### 2.6 TEG for Impressed Current Cathodic Protection of Steel Bars

**Figure 6:**
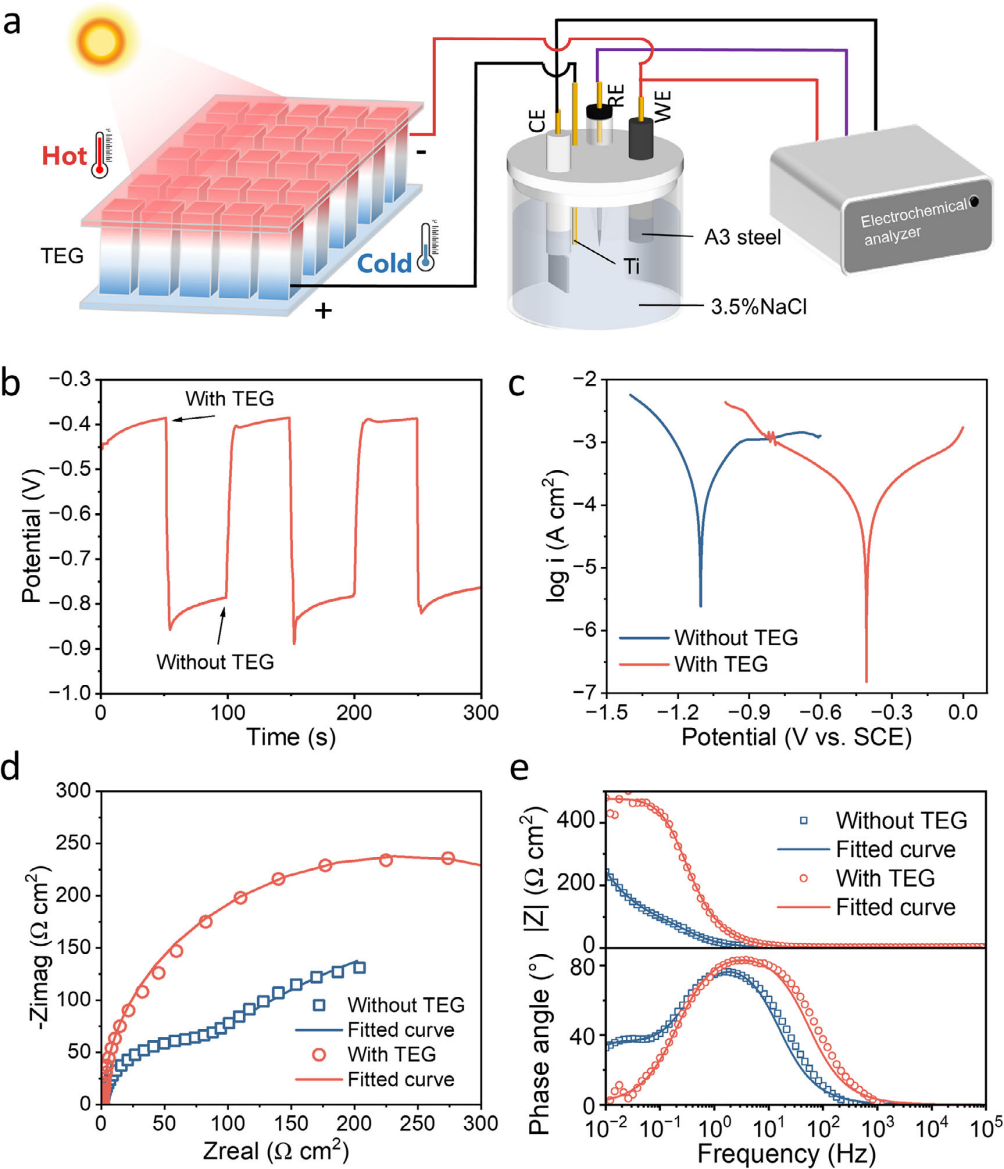
Application of TEG for the self‐powered cathodic protection system of rebar. a) Schematic diagram of TEG for cathodic protection, b) Open circuit voltage drops of rebar coupled with TEG, c) Tafel curve of rebar with and without TEG, d) Nyquist plots of rebar with and without TEG, e) Impedance mode value and phase Angle of EIS results.

The thermoelectric energy harvesting capability of cementitious composites demonstrates their potential for self‐powered impressed current cathodic protection (ICCP) systems in reinforced concrete structures. Under a mild thermal gradient (ΔT = 50 K), the TEG maintains 98.2% current persistence during 20‐h continuous operation (Figure S28, Supporting Information). **Figure** **6**a shows the TEG‐powered ICCP configuration. A3 steel (10 mm in diameter and 50 mm in length) was pre‐embedded in a 20 mm diameter cement paste specimen as the working electrode. During ICCP operation, the TEG anode electrode is connected to the auxiliary anode (Titanium mesh), while the cathode couples to the A3 steel. When TEG operates, the electron flow starts from the negative electrode of the TEG, passes through the wire, and is directly transported to the A3 steel to form a closed circuit. Electrochemical characterization revealed significant corrosion mitigation upon TEG activation, with the open‐circuit potential (OCP) shifting from ‐386 to ‐851 mV (vs SCE) (Figure 6b). The potential shift indicates continuous electron migration from the rebar to the material, satisfying the cathodic protection standard (ASTM C876 requires OCP > ‐0.25 V). Tafel analysis (Figure 6c) further confirms protection efficacy, showing both reduced corrosion current density *i*_corr_ from 1.63 to 0.86 µA cm^−2^ and decreasing corrosion rate from 7.42 to 3.93 mm a^−1^ with a reduction ratio of 47.04% (Table S8, Supporting Information).

EIS analysis was conducted to further investigate the rebars' corrosion resistance (Figure 6d). The equivalent circuit is shown in Figure S29 (Supporting Information), where R_s_ is the solution resistance, R_f_ and CPE_f_ denote the membrane resistance and membrane capacitance of the passivation film/corrosion product layer, respectively, and R_ct_ and CPE_dl_ denote the charge‐transfer resistance and double‐layer capacitance, respectively.^[^ ^22^ ^]^ The equivalent circuit component values are listed in Table S9 (Supporting Information). The Nyquist plot shows a significant increase in the semicircular arc after TEG protection, which indicates an increase in charge transfer resistance (R_ct_) due to the suppression of the corrosion reaction (Figure 6d). Bode plot (Figure 6e) shows the phase angle curve shifting toward higher frequencies, and the low‐frequency impedance modulus |Z| increased from 244 to 480 Ω cm^2^. This phenomenon reflects the enhanced interfacial reaction resistance. Further investigation presented in Figures S30–S32 (Supporting Information) explores the effects of corrosion exposure duration, environmental conditions, and steel substrate types on the cathodic protection performance of the TEG. Corresponding Tafel curve parameters and EIS fitting results are summarized in Tables S10 and S11 (Supporting Information). The results indicate that both EIS profiles and Tafel curves of A3 steel progressively stabilize as the duration of cathodic protection increases from 1 to 7 days.

## 3 Conclusion

This study proposes and fabricates a CNT/PEDOT: PSS(PP)/ Bi₀._5_Sb₁._5_Te_3_(BST) cement‐based composite with high thermoelectric performance, superior mechanical strength, and long‐term durability. The addition of PP effectively inhibits the agglomeration of functional fillers in the cementitious system through *π–π* stacking interactions and hydrogen bonding, thereby enhancing the uniform dispersion of the components. The interfacial barriers formed between PP and BST synergistically elevate electrical conductivity to 1.81 S m^−1^, achieve a S of 450 µV K^−1^, and reduce thermal conductivity to 0.458 W m^−1^ K^−1^. This results in a significant 27% increase in the power factor, reaching 41.8 µW cm^−1^ K^−2^. Furthermore, the ternary composite significantly enhances mechanical strength and chloride penetration resistance. After 60 days of exposure to extreme temperature, the optimal sample retains over 8% improvement in mechanical strength and anti‐chloride permeability compared to the control group. The integration of 30 units into a TEG yields a voltage output of 0.89 V and a maximum power of 26.1 µW at a temperature difference of 75 K, with less than 8% performance decay after 20 h of continuous operation. This setup successfully powers a commercial bulb. When applied as a self‐powered cathodic protection system, the composite reduces steel corrosion current density and corrosion rate by over 47%, while increasing electrochemical impedance modulus by more than 50%. This work provides a prospective solution for sustainable self‐powered systems and infrastructure applications.

## Conflict of Interest

The authors declare no conflict of interest.

## Supporting information



Supporting Information

## Data Availability

The data that support the findings of this study are available from the corresponding author upon reasonable request.
