# Supplementary material for: PEDOT: PSS‐Enabled CNT and Bi0.5Sb1.5Te3 Interface Microengineering for Integrated Thermoelectric Energy Harvesting and Corrosion Protection in Cementitious Composite
Source: Adv Sci (Weinh). 2025 Aug 19;12(42):e08424. doi: 10.1002/advs.202508424 (PMC12622541; doi:10.1002/advs.202508424)
Supplement: Supplementary file 1 — Supporting Information [file ADVS-12-e08424-s001.docx]

Supporting Information

**PEDOT: PSS-Enabled CNT and Bi₀.₅Sb₁.₅Te₃ Interface Microengineering for Integrated Thermoelectric Energy Harvesting and Corrosion Protection in Cementitious Composite**

*Chen Zhang*^1^, *Yingwu Zhou*^1^, *Feng Xing*, *Menghuan Guo**, *Jiawei Wu*, *Junfeng Wang, Yinghui Wu**

1 These authors contributed equally to this work

* These authors contributed equally as co-corresponding authors

C. Zhang, Y. Zhou, F. Xing, M. Guo, J. Wu, J. Wang, Y. Wu

Guangdong Provincial Key Laboratory of Durability for Marine Civil Engineering, Shenzhen University, Shenzhen 518060, P.R. China

E-mail: [menghuan.guo@szu.edu.cn](mailto:menghuan.guo@szu.edu.cn)

F. Xing, J. Wang, Y. Wu

School of Mechanics and Construction Engineering, Jinan University, Guangzhou 51063, P.R. China

E-mail: [yinghuiwu@jnu.edu.cn](mailto:yinghuiwu@jnu.edu.cn)

# Experimental Section

***Materials:*** The ordinary Portland cement (P.O 42.5) was purchased from Shenzhen Shouxin Cement Co., Ltd., China. The chemical composition of cement is summarized in Table S12. Multi-walled carbon nanotubes (MWCNTs, purity ≥ 95 wt%, diameter 1–3 nm) were obtained from Suzhou Tanfeng Technology Co., Ltd., China, with additional details provided in Table S13. Bi_0.5_Sb_1.5_Te_3_(BST) was self-synthesized as nano-powders in the laboratory. The PEDOT: PSS aqueous solution (Clevios™ 4083, solid content ~1.50 wt%) was sourced from Heraeus Materials Technology Shanghai Ltd. Sodium dodecylbenzene sulfonate (SDBS) was supplied by Sinopharm Chemical Reagent Co., Ltd., China. Tributyl phosphate (TBP) employed as a defoamer, was purchased from Shanghai Macklin Biochemical Co., Ltd. A polycarboxylate-based high-performance water reducer (HPWR-S, water reduction ratio >25%) used to adjust the fluidity of the cement paste, was provided by Shanxi Feike New Material Technology Co., Ltd. Chemical reagents including Bi(NO₃)₃·5H₂O, SbCl₃, TeO₂, NaOH, and N₂H₄ were acquired from Shanghai Aladdin Biochemical Technology Co., Ltd. All experiments utilized deionized water.

***Preparation of CNT/PEDOT: PSS /Bi_0.5_Sb_1.5_Te_3_ Cement-based Composite:*** The Bi_0.5_Sb_1.5_Te_3_ (BST) nano-powders were prepared by the solvothermal method. Specifically, 0.5 mmol Bi(NO₃)₃·5H₂O, 1.5 mmol SbCl₃, 3 mmol TeO₂, and 5 mol/L N₂H₄ were dissolved in 20 mL of ethylene glycol. Adjust the pH of the mixture to 11 using a NaOH solution, and then transfer the solution to a Teflon-lined autoclave. The reaction proceeded at 210°C for 12 hours under a nitrogen atmosphere. After cooling to room temperature, the product was collected via centrifugation, washed several times with deionized water and absolute ethanol, dried at 60°C and ground into fine powder to obtain the BST nanoparticles. The SEM-EDS characterization of BST nanoparticles is shown in Figure S2. The MWCNTs were acid-treated to remove surface impurities, dried for 24 h and ground for 4 h to obtain the mixture. The mixture was ultrasonically dispersed in an aqueous solution containing SDBS for 30 minutes. Subsequently, BST and PEDOT: PSS aqueous solution were added, followed by further ultrasonication for 30 minutes at 20°C. Next, deionized water (water-to-cement ratio is 0.4), TBP (0.2 mL), and water reducer (1 g) were incorporated into the cement composite mixture. The mixture was stirred at 65 rpm and 125 rpm for 4 min. The cement composite slurry was poured into rectangular polytetrafluoroethylene molds (20×20×20 mm³, 10×10×30 mm³). Four groups of specimens (CNT, CNTPP, CNTBST and CNTBSTPP) were prepared. After curing in a standard curing chamber (temperature was 20 ±2°C and humidity was 90 ±5%) for 7 d, the specimens were dried in an oven at 60°C for 24 h.

***Characterizations:*** A Zeta potential analyzer (Malvern Zetasizer Nano) was used to characterize the stability of the suspensions. Dynamic light scattering (DLS) was employed to determine the average particle size and size distribution. Scanning electron microscopy (SEM, Thermo Fisher Apreo 2) coupled with energy-dispersive X-ray spectroscopy (EDX-LE Plus) was utilized to analyze the morphological features and chemical composition of the composite surfaces. Phase composition and mineralogical analysis were conducted using an X-ray diffraction (XRD) spectrometer (Bruker D8 Advance) with a scanning step of 0.02° over a 2θ range of 10° to 70°. Raman spectroscopy (Renishaw inVia 2000) was applied to investigate the phase composition and carbon states of the composites. Functional group interactions within the composites were observed using Fourier-transform infrared spectroscopy (FTIR, Bruker ALPHA II). The chemical composition and valence state information of the composites were identified by X-ray photoelectron spectroscopy (XPS, Thermo Fisher ESCALAB 250 Xi). Ultraviolet-visible (UV-Vis) reflectance spectroscopy (PerkinElmer Lambda 750) was performed to measure the optical reflectance spectra of the materials*.*

***Measurements of TE, Mechanical and Durable Performances:*** The cement composites were coated with conductive silver paste at both ends and connected to the testing setup via adhered copper sheets to measure the electrical conductivity (σ) and Seebeck coefficient (S). The tight bonding between the specimens and copper sheets through the conductive silver paste minimized the influence of contact resistance on the test results. A digital multimeter (Fluke 8808A) was used to measure the electrical conductivity and potential of the composites. The temperature gradient across the sample was recorded using a temperature probe, and the ratio of the potential difference to the temperature gradient yielded the S. The interfacial charge transfer resistance of the composites was analyzed by electrochemical impedance spectroscopy (EIS) and Cyclic voltammetry (CV) using an electrochemical analyzer (CHI 660E).

The carrier concentration ($\eta_{H}$) and carrier mobility ($\mu_{H}$​) were calculated using the equations $\eta_{H}=1/\left(eR_{H}\right)$ and $\mu_{H}=\sigma R_{H}$​, respectively, where Hall coefficient ​ (R_H_) was obtained at room temperature using a Physical Property Measurement System (PPMS-9, Quantum Design) under a magnetic field ranging from -5 to 5 T. The total thermal conductivity (κ_tot_) of the composites was determined via the laser flash method (Netzsch LFA 467) on sheets with dimensions of 10 × 10 × 1 mm³, and the heat capacity (C_p​_) was derived using the Dulong-Petit law. The electronic thermal conductivity (κ_e_) was calculated using the Wiedemann-Franz law ($\kappa_{e}=L\sigma T$).

To evaluate the electrothermal performance, the composites were powered by a DC power supply (RXN-305D, Zhaoxin, China). Heating tests were conducted at an ambient temperature of 25°C. Infrared thermal images and surface temperature profiles of the samples were acquired using an infrared thermal camera (Fluke Tis55+).

The compressive strength and flexural strength of the cement composites were evaluated after 7 days of curing using a computer-controlled universal testing machine (Droide YAW-300B). The flexural strength was determined by testing specimens with dimensions of 20 × 20 × 80 mm³ at a constant loading rate of 0.02 mm min⁻¹. The compressive strength was assessed by compressing cubic specimens (20 × 20 × 20 mm³) at a loading rate of 1.2 mm min⁻¹. The flexural and compressive strength results were reported as the arithmetic mean of three individual samples.

A concrete chloride ion diffusion coefficient tester (RCM-6, Beijing Durability Weiye Technology Co., Ltd.) was employed to evaluate the chloride penetration resistance of the composites under different temperature conditions.

***TEG Preparation and Output Performance Measurements:*** The thermoelectric generator (TEG) had an overall dimension of 10 cm × 10 cm^2^ and consisted of 30 p-type legs (10 × 10 × 30 mm³) connected in series. Copper wires and silver paste were used to serially interconnect the thermoelectric legs, with copper foil serving as electrodes at the junctions. The performance of the TEG was evaluated using a custom-built temperature gradient system. The cold-side temperature (Tc) was maintained near room temperature via forced water circulation, while the hot-side temperature (Th) was elevated to the desired testing level using a heating stage. A COMSOL Multiphysics model of the TEG with identical dimensions was established to simulate the output power (*P*) and heat flow at the cold side (*Q*). The thermoelectric energy conversion efficiency (𝜂) was calculated as$\eta=P/\left(Q+P\right)$.

Additionally, to investigate the impressed current cathodic protection (ICCP) for steel reinforcement in concrete, open-circuit potential (OCP), electrochemical impedance spectroscopy (EIS), and Tafel polarization curves were measured using the aforementioned electrochemical workstation in a 3.5 wt% NaCl solution. Cement specimens embedded with Q235 steel (10 mm diameter) were prepared as the working electrode. Prior to electrochemical testing, the working electrode was immersed in 3.5 wt% NaCl solution for 2 h to achieve potential and environmental stabilization.

The calculation equation of band gap width (*E*g)[9]：

$\left(\alpha h\nu\right)^{1/2}=B\left(h\nu-E_{g}\right)$ (S1)

where ℎ is Planck's constant, 𝜈 is the frequency of light, and 𝛼 is the absorption coefficient.

The calculation equation of electron thermal conductivity (*κ*_e_)：

*$\kappa_{\text{e}}=L\sigma T$* (S2)

where *L* is the Lorenz factor, obtained from the equation *L*=1.5+exp (-|S|/116) ×10^-8^, V^2^ K^-2^. [10] *σ* is the electric conductivity, S m^-1^. *T* is the absolute temperature, K. The carrier concentration inside semiconductors and insulators is much lower than in metals, so their electronic thermal conductivity is usually smaller, and the heat conduction is mainly contributed by phonons.

The calculation equation of surface temperature error ($\varepsilon_{T}$):

$\varepsilon_{T}=\frac{T_{\max}-T_{\min}}{T_{avg}}\times100\%$ (S3)

where *T_max_* is the highest test temperature, ℃. *T_min_* is the lowest test temperature, ℃. *T_avg_* is the average temperature of surface, ℃.

The calculation equation of output power (*P*):

$P=I^{2}R_{L}=\left(\frac{V_{oc}}{R_{in}+R_{L}}\right)^{2}R_{L}$ (S4)

where *I* is the current through the load, A. *R_L_* is the load resistance, Ω. *R_in_* is the internal resistance of the device, Ω. *V_OC_* is the open circuit voltage (measured *V_TEG_*) as *R_L_* approaches infinity, V.


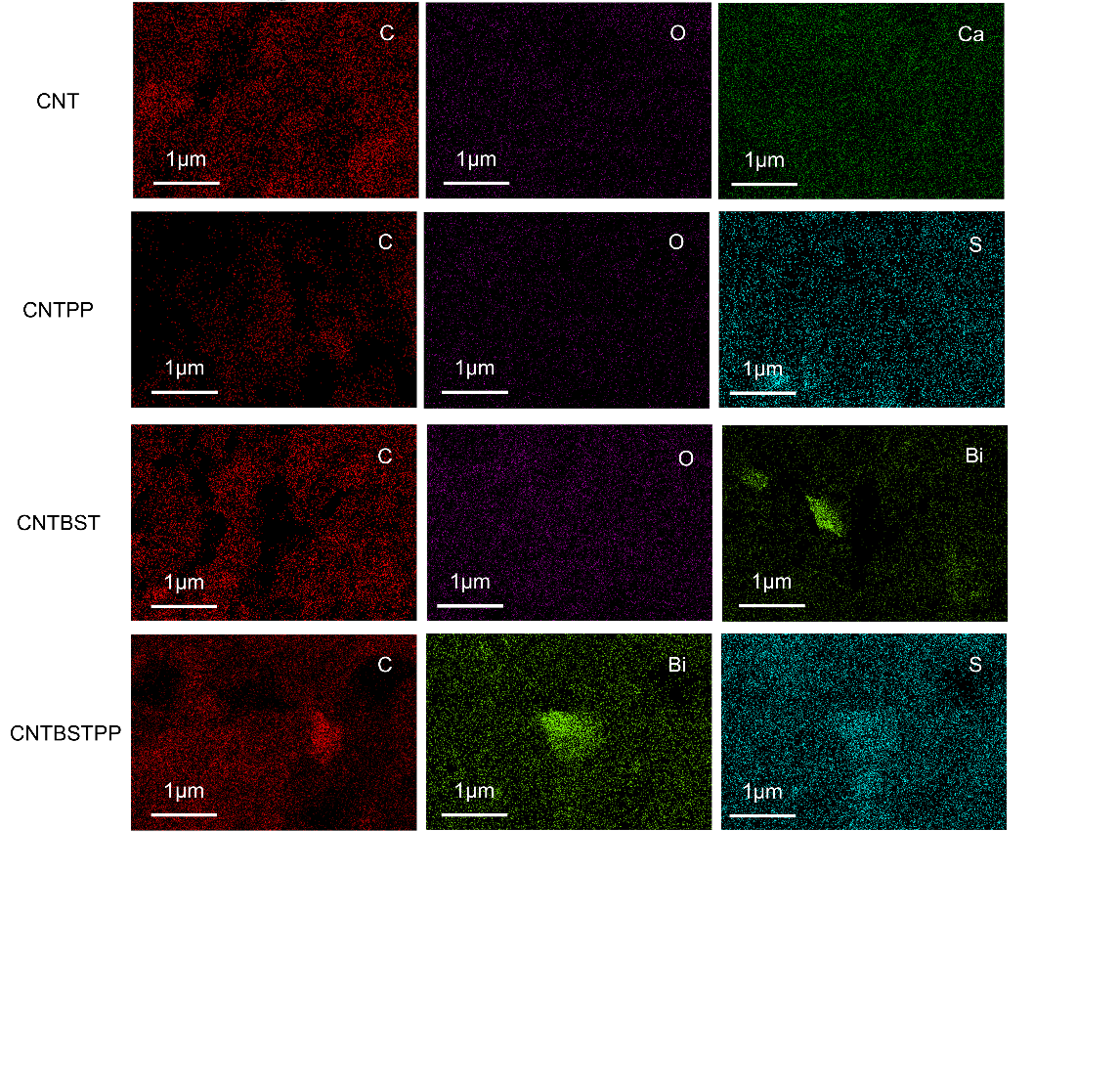


**Figure S1** EDS results of CNT/PEDOT: PSS (PP)/ Bi_0.5_Sb_1.5_Te_3_ (BST) cement-based materials.


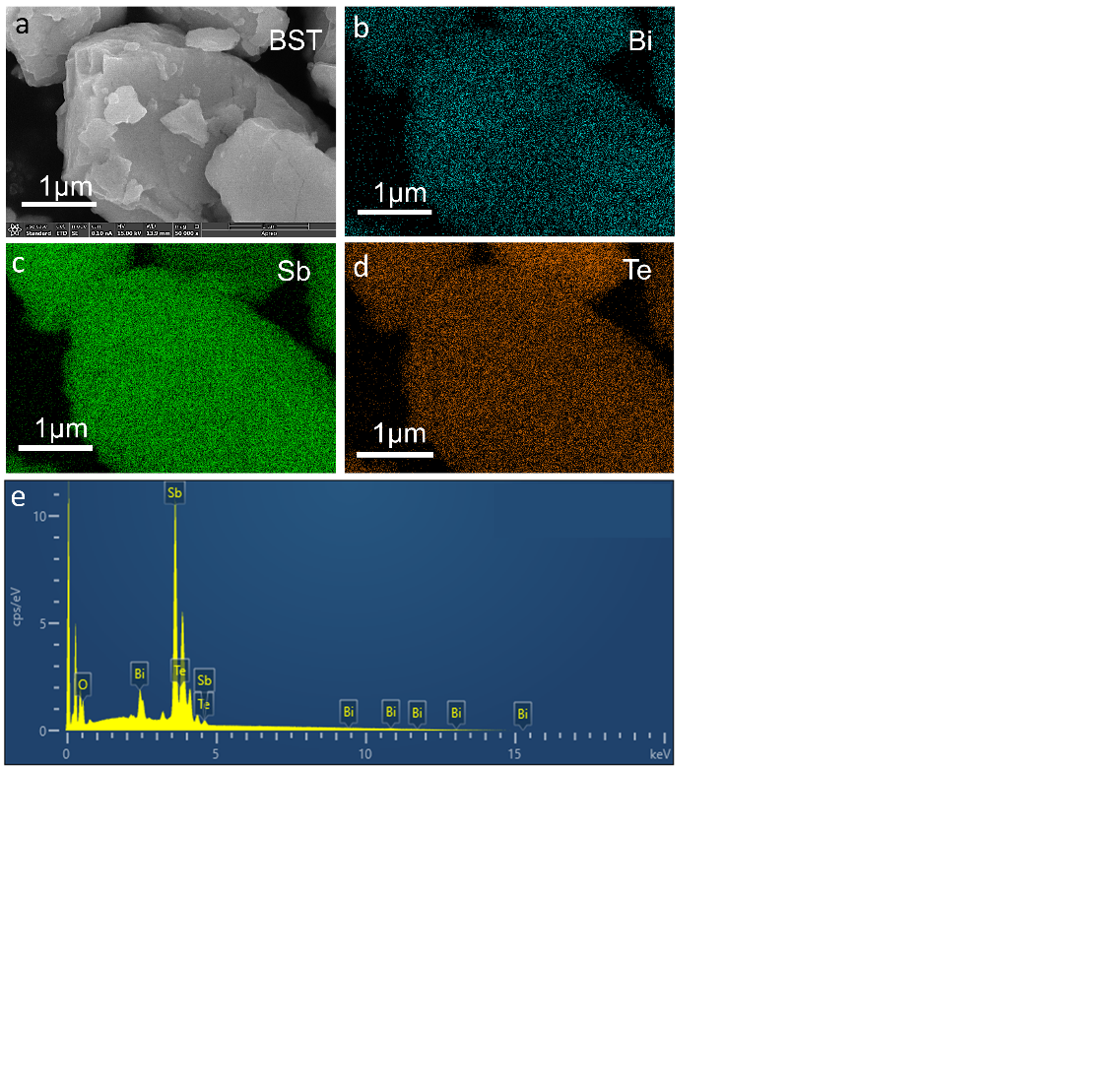


**Figure S2** SEM-EDS characterization of BST. (a) SEM of BST. EDS of BST: (b) Bi element. (c) Sb element. (d) Te element. (e) Distribution diagram of element.

**
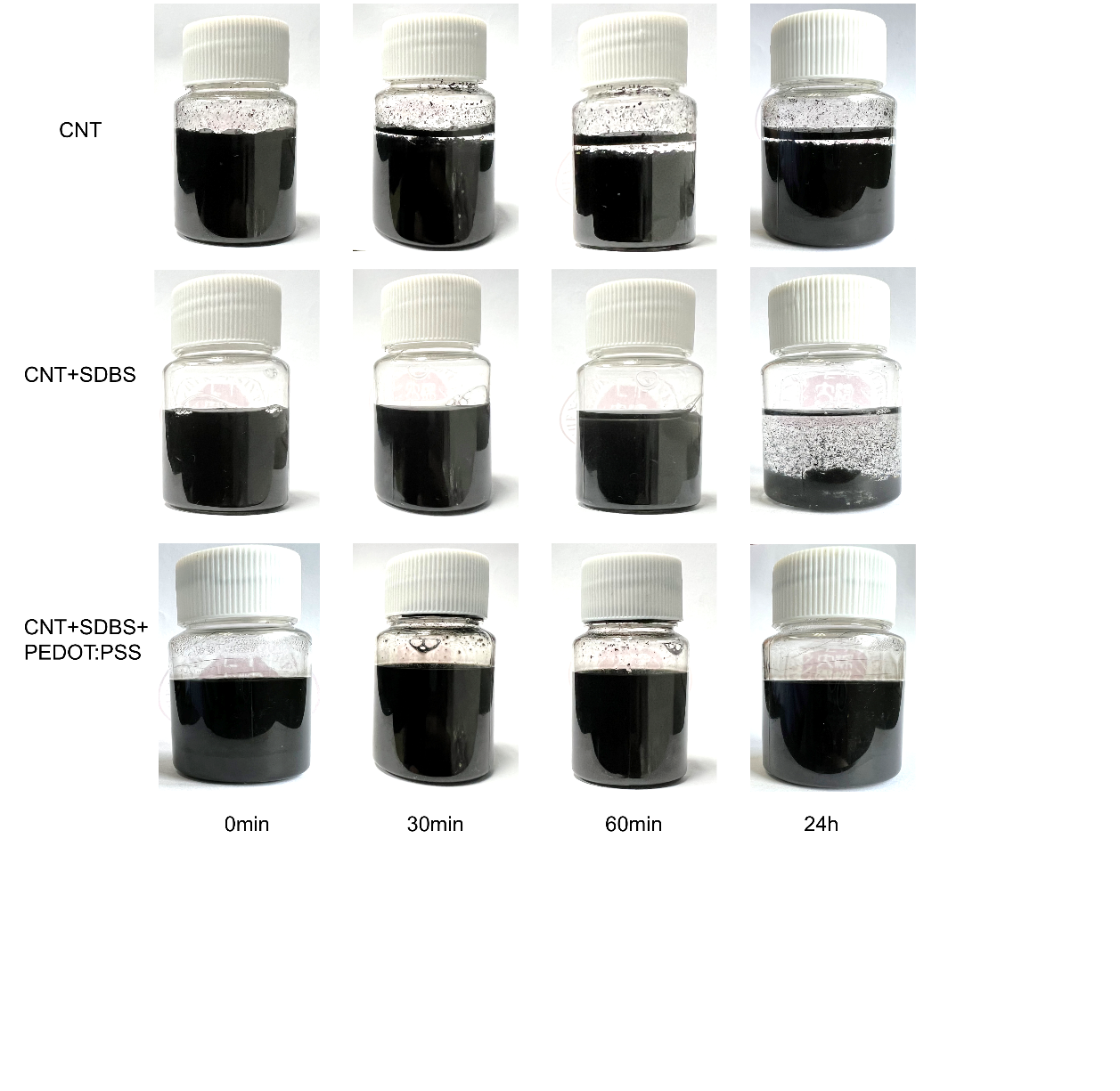
**

**Figure S3** Visual settlement test of CNT in different aqueous solutions.


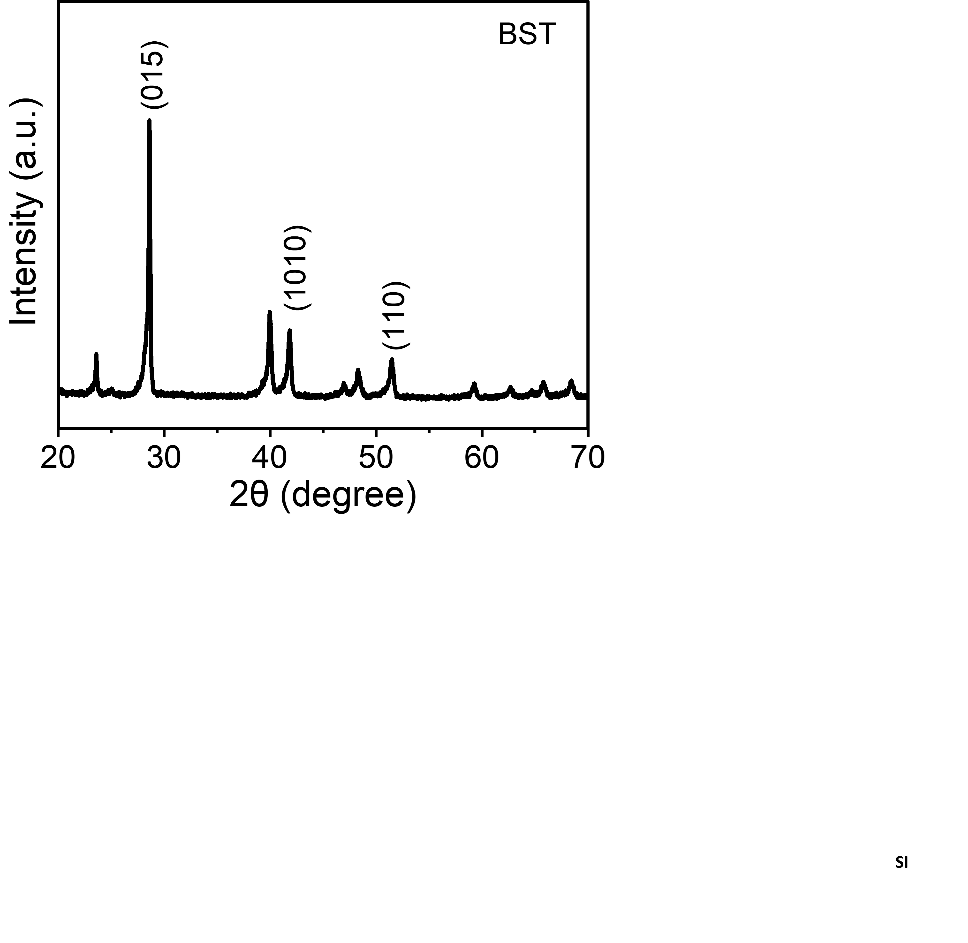


**Figure S4** XRD pattern of BST.


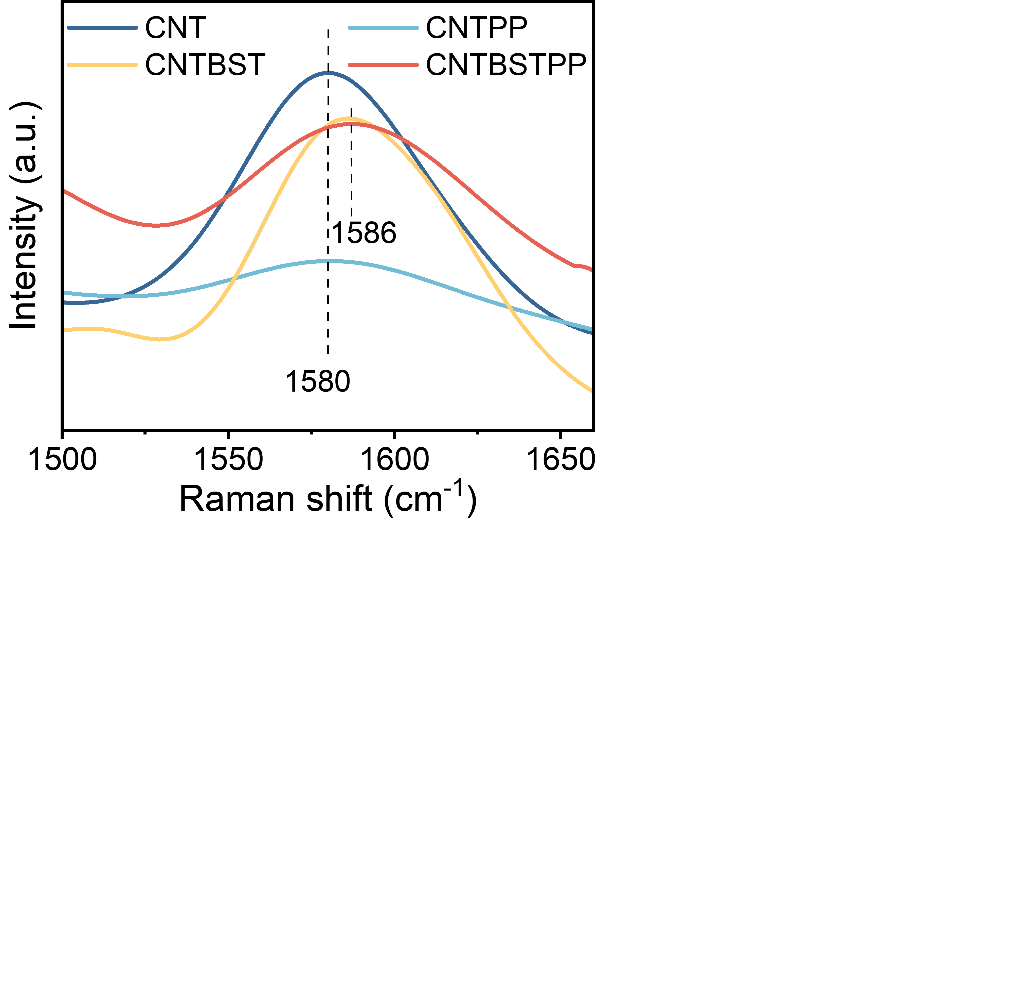


**Figure S5** Raman spectra at 1580 cm^-1^ of CNT/PP/ BST cement-based materials.


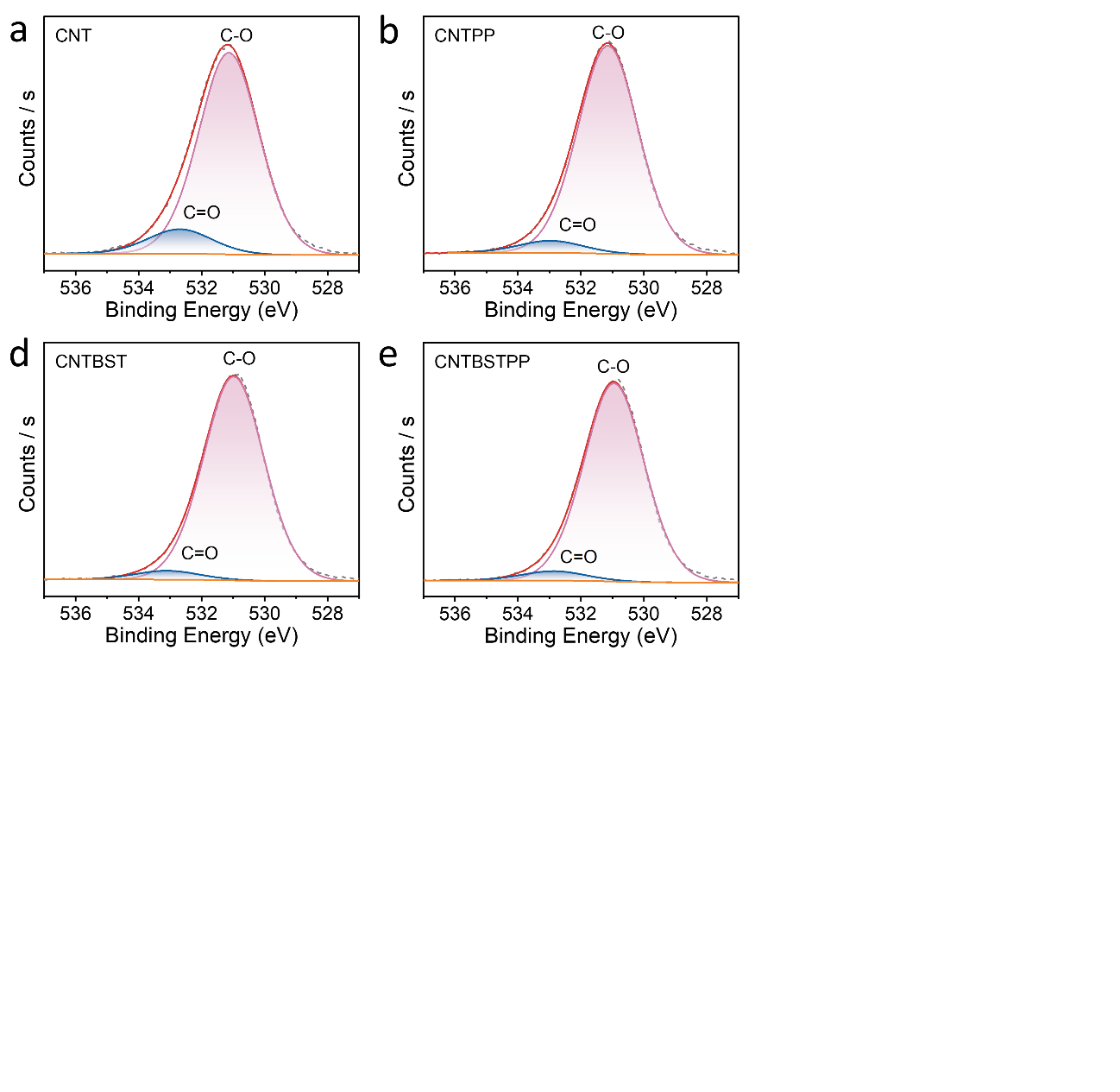


**Figure S6** O 1s peak of XPS spectra. (a) CNT. (b) CNTPP. (c) CNTBST. (d) CNTBSTPP.


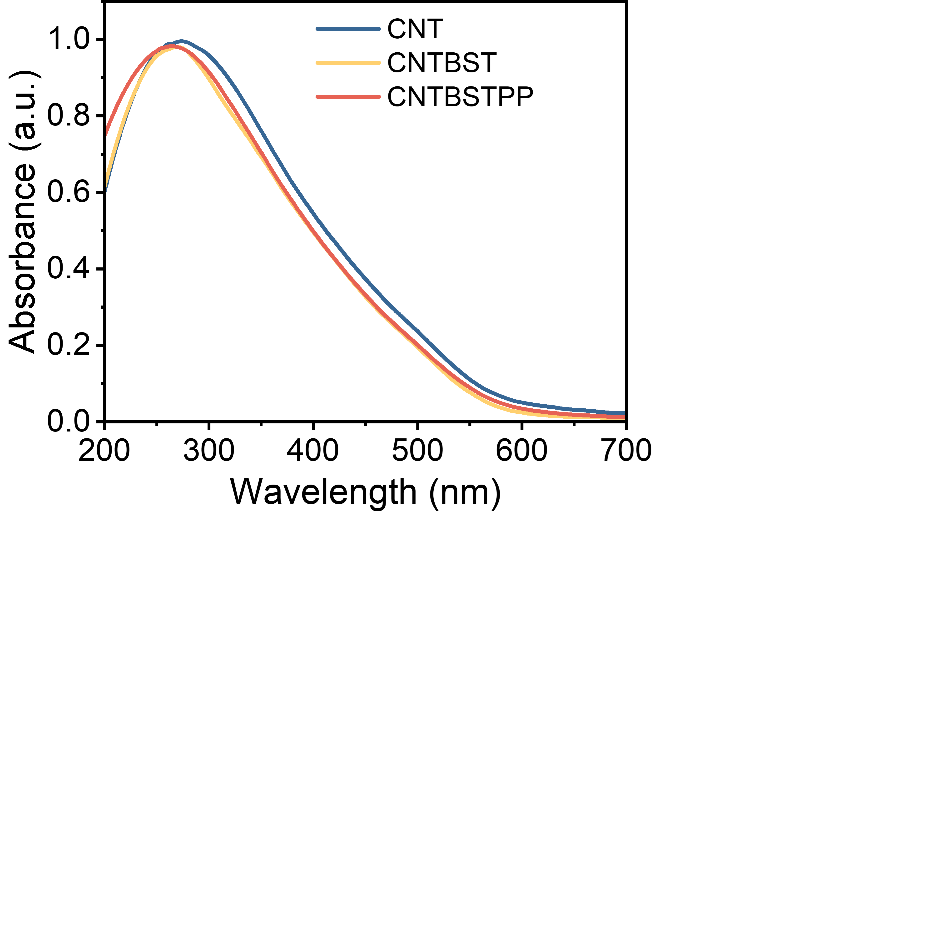


**Figure S7** UV-vis spectrum of CNT/PP/BST cement-based materials.


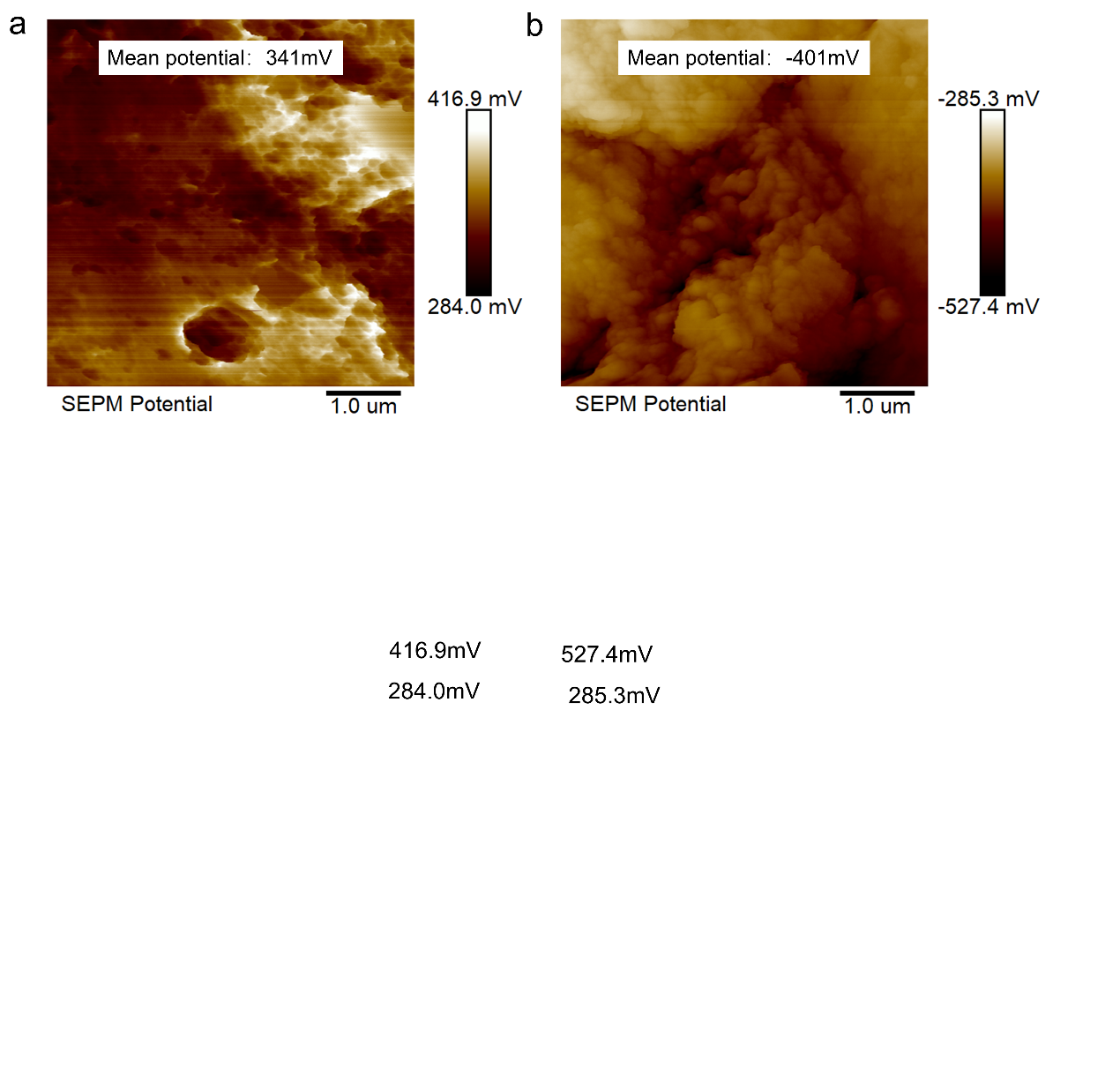


**Figures S8** The KPFM results of (a) CNT and (b) CNTBSTPP.


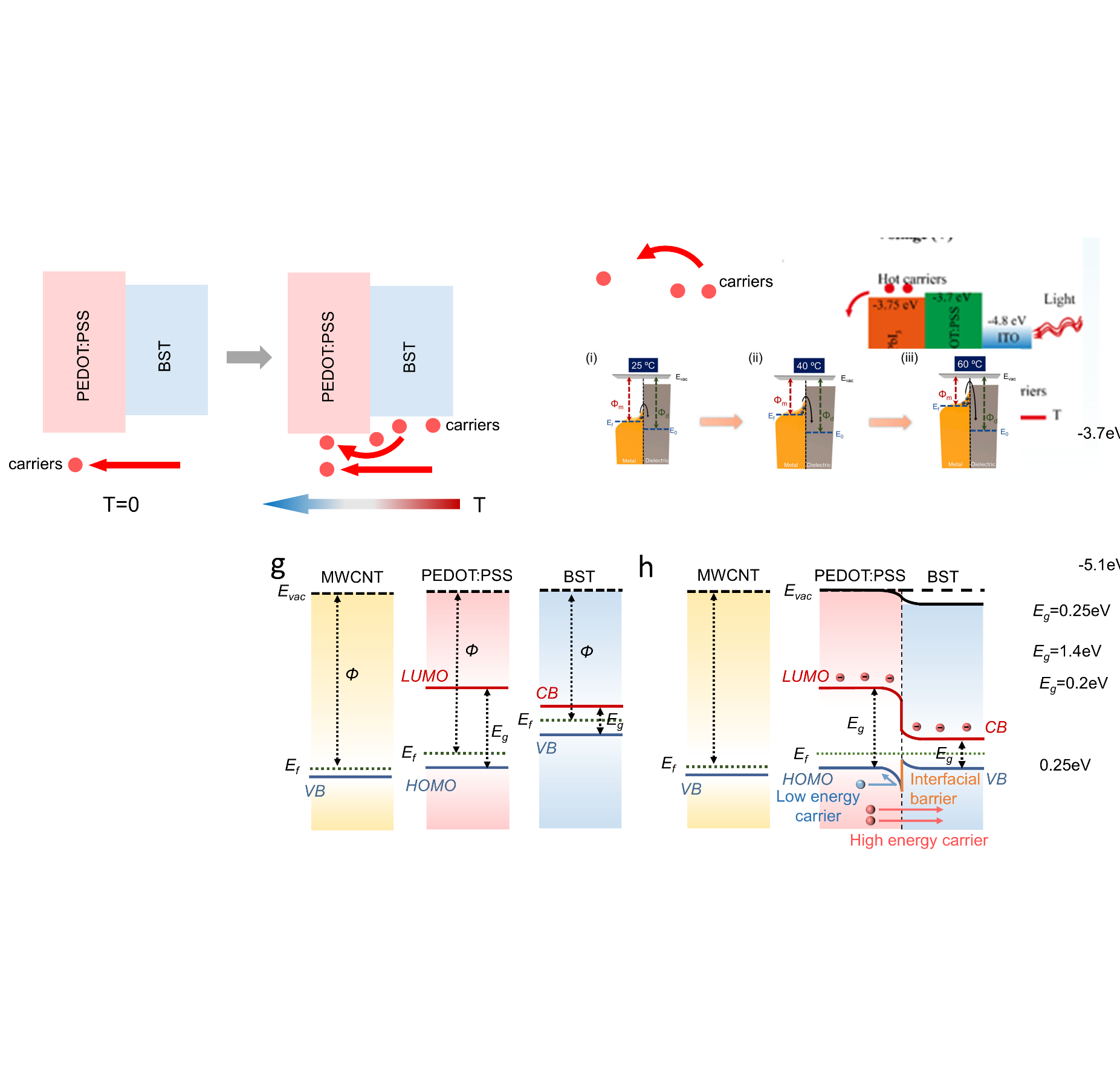


**Figure S9** The temperature-dependent carriers transfer mechanism of PEDOT: PSS/BST interface.


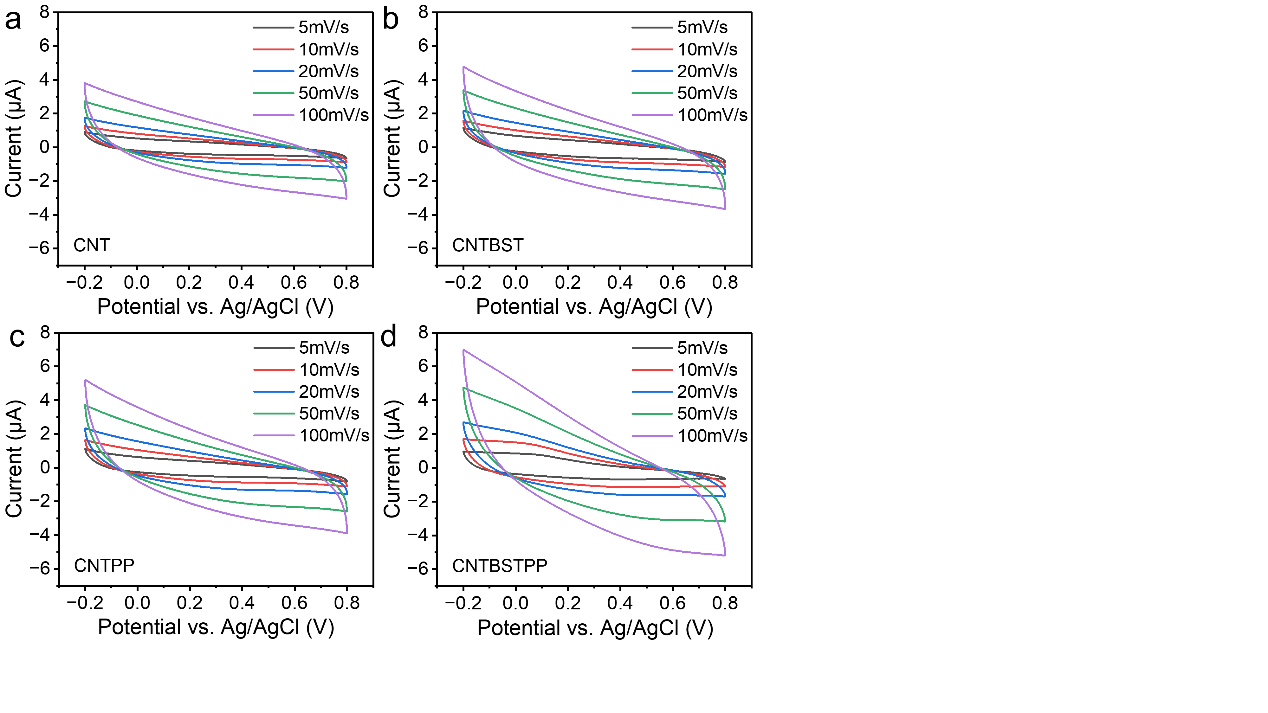


**Figure S10** CV test results. (a)CNT. (b)CNTBST. (c) CNTPP. (d) CNTBSTPP.


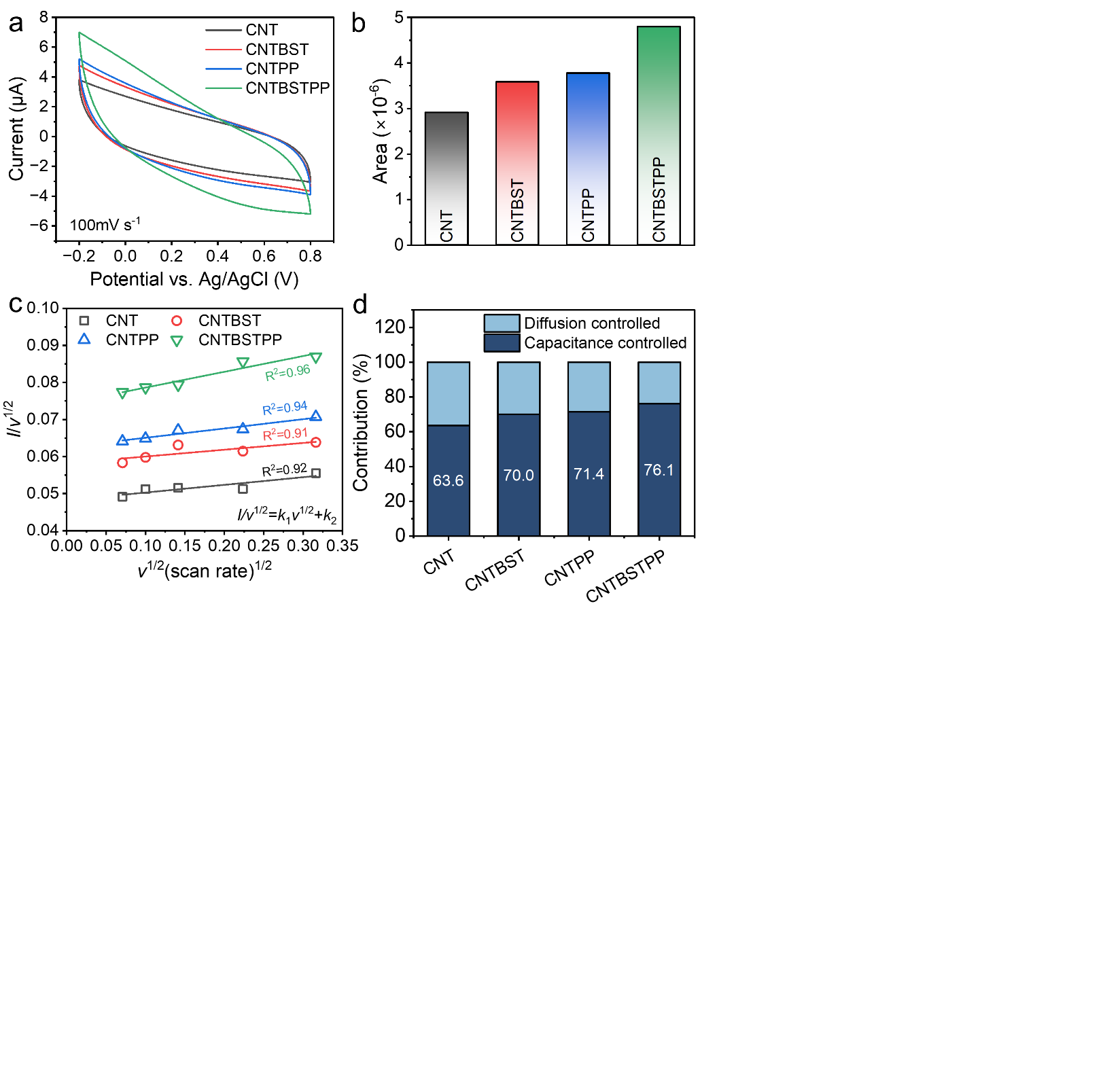


**Figure S11** (a) CV test results of 100 mV s^-1^. (b) Integral area of CV. (c) Plot of I/v^1/2^ vs v^1/2^. (d) Calculation results of capacitance contribution ratio.


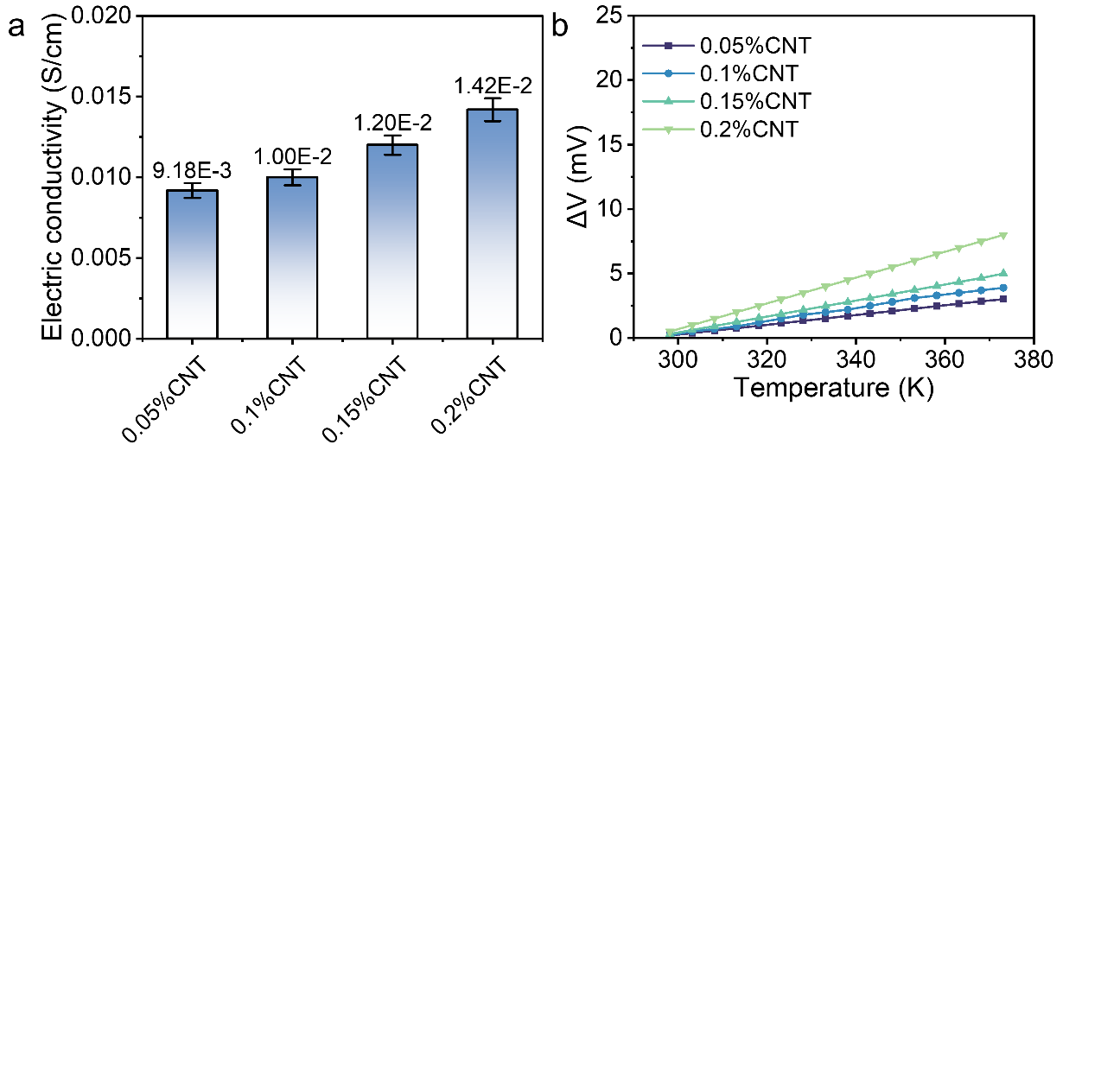


**Figure S12** Conductivity and voltage(∆V)-temperature(T) diagram of cement-based materials with different CNT content. (a) Conductivity. (b) ∆V-T diagram.


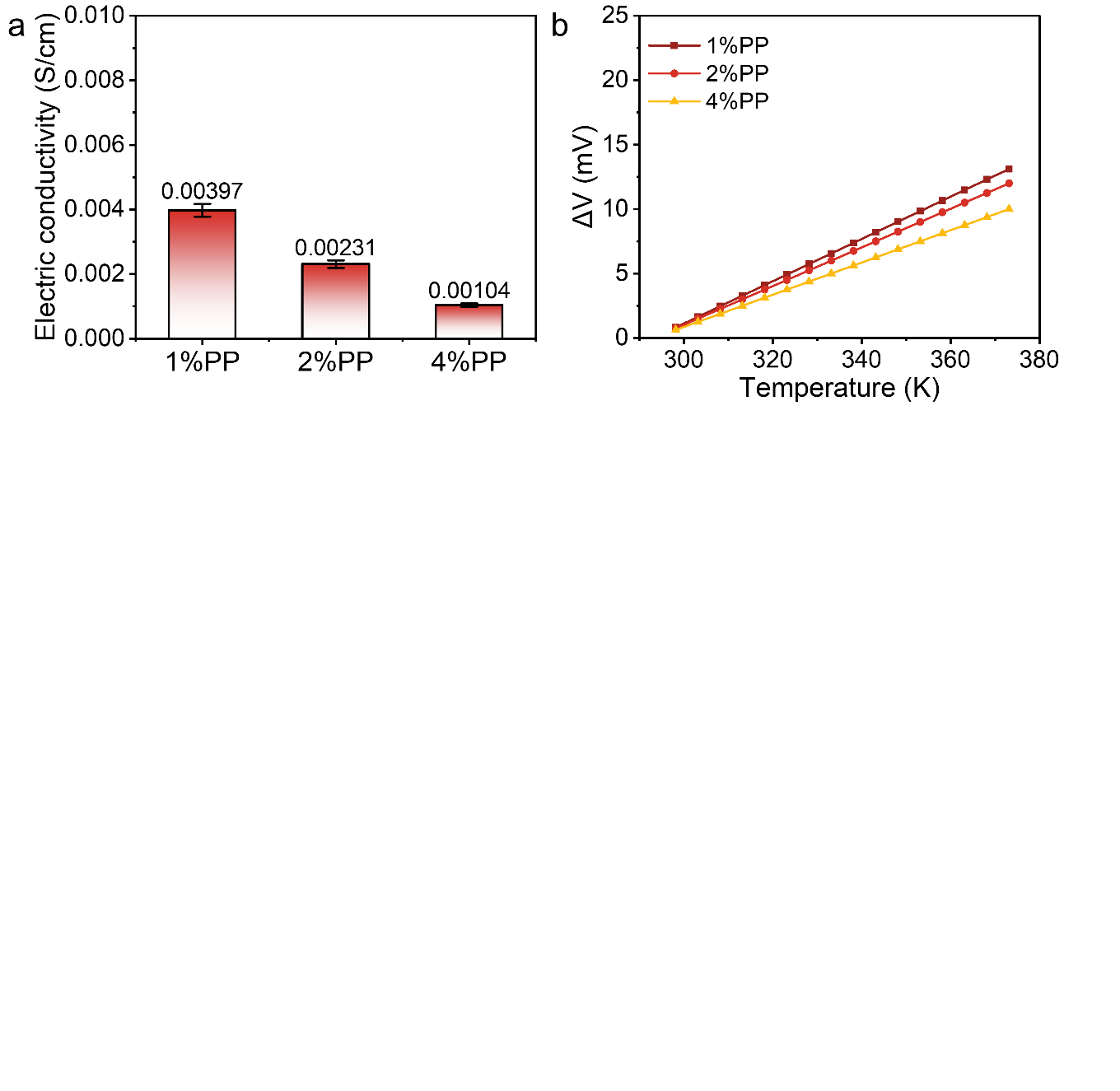


**Figure S13** Conductivity and ∆V-T diagram of cement-based materials with different PP content. (a) Conductivity. (b) ∆V-T diagram.


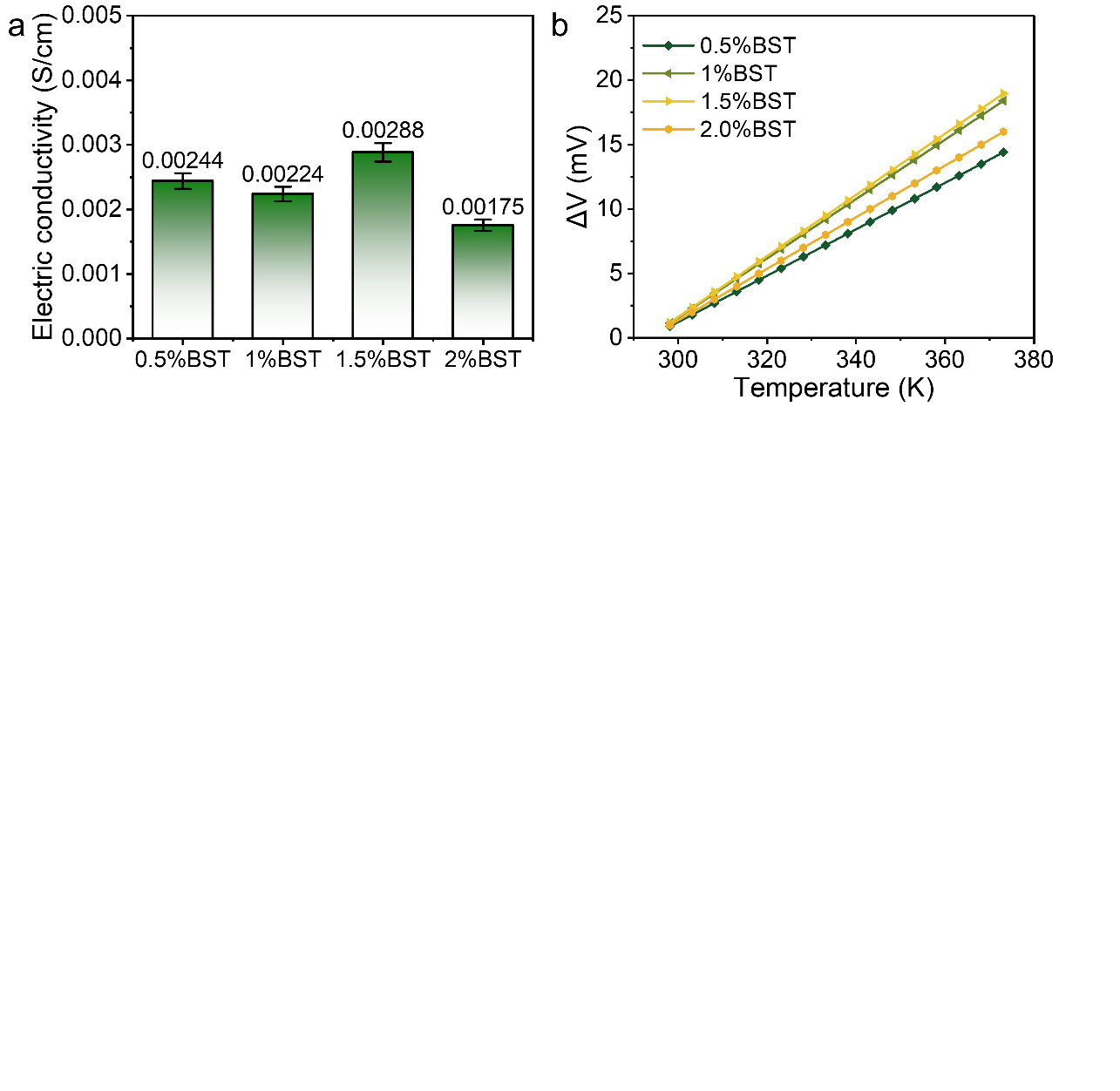


**Figure S14** Conductivity and ∆V-T diagram of cement-based materials with different BST content. (a) Conductivity. (b) ∆V-T diagram.

**Figure S15** FTIR spectra after 60-day curing.

**Figure S16** Tauc plots of UV-Vis spectra after 7-day and 60-day curing.

**
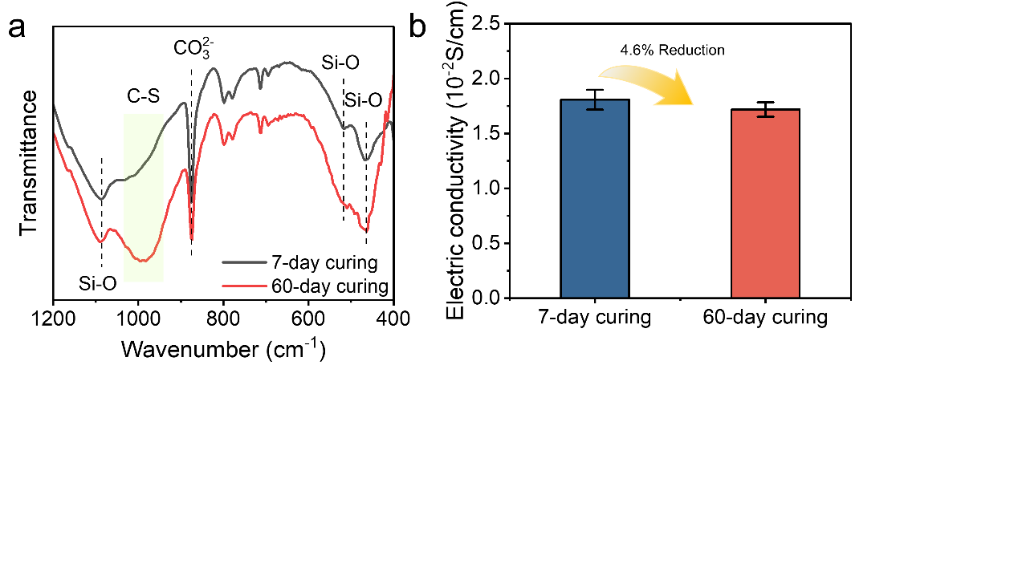
**

**Figure S17** Electric conductivity after 60-day curing.


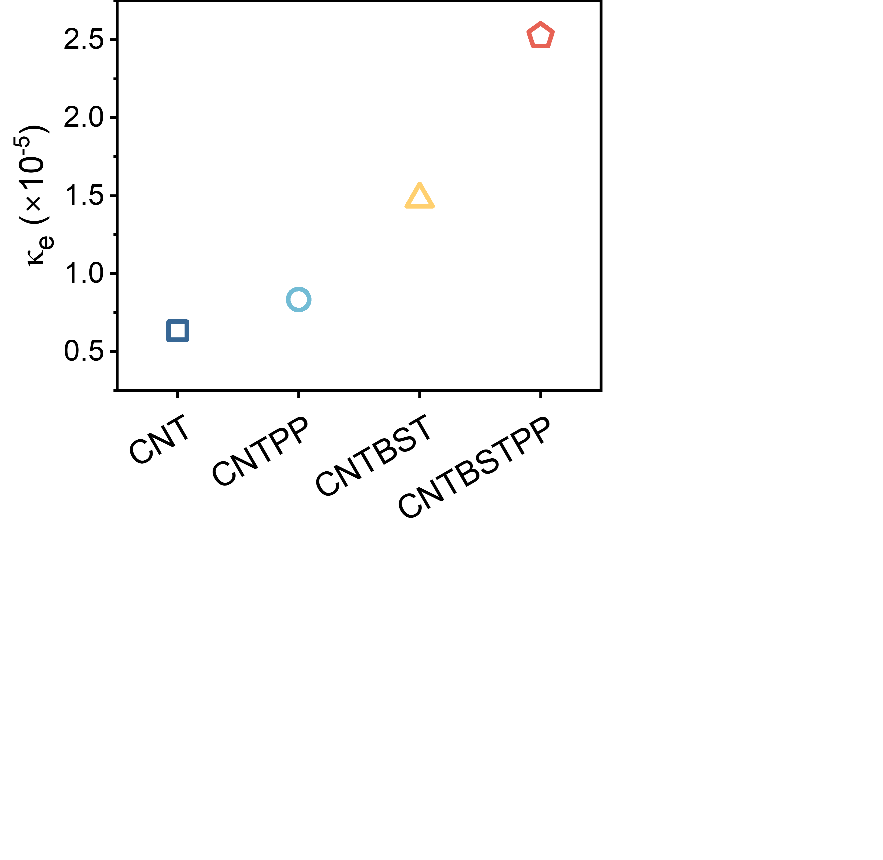


**Figure S18** Electrons thermal conductivity (κ_e_) of CNT/PP/BST cement-based materials.


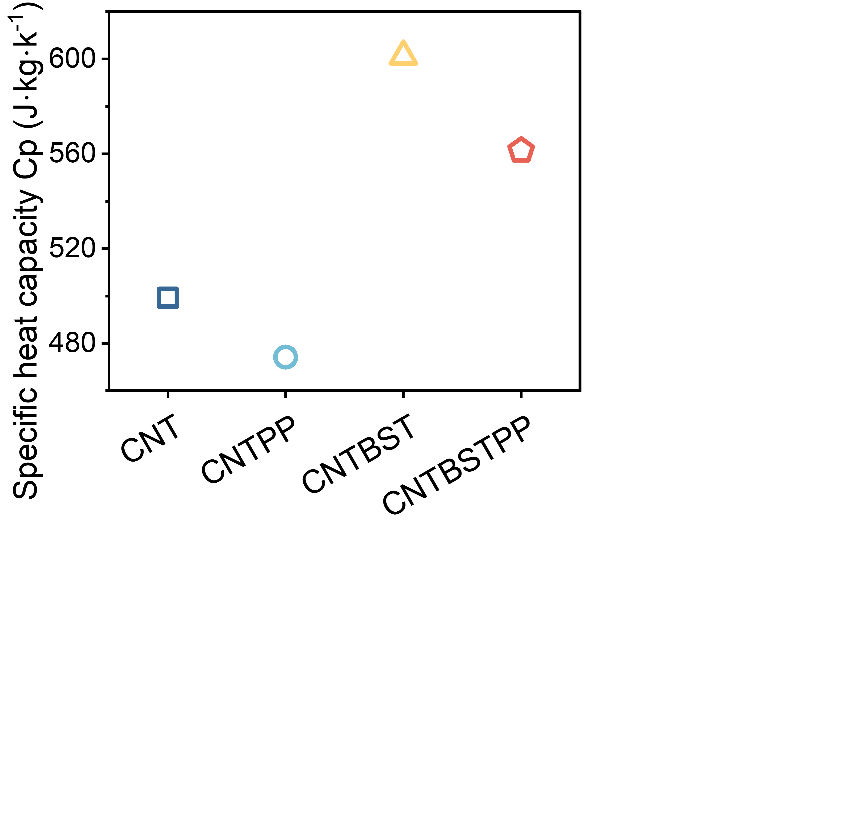


**Figure S19** Specific Heat Capacity (C_p_) of CNT/PP/BST cement-based materials.


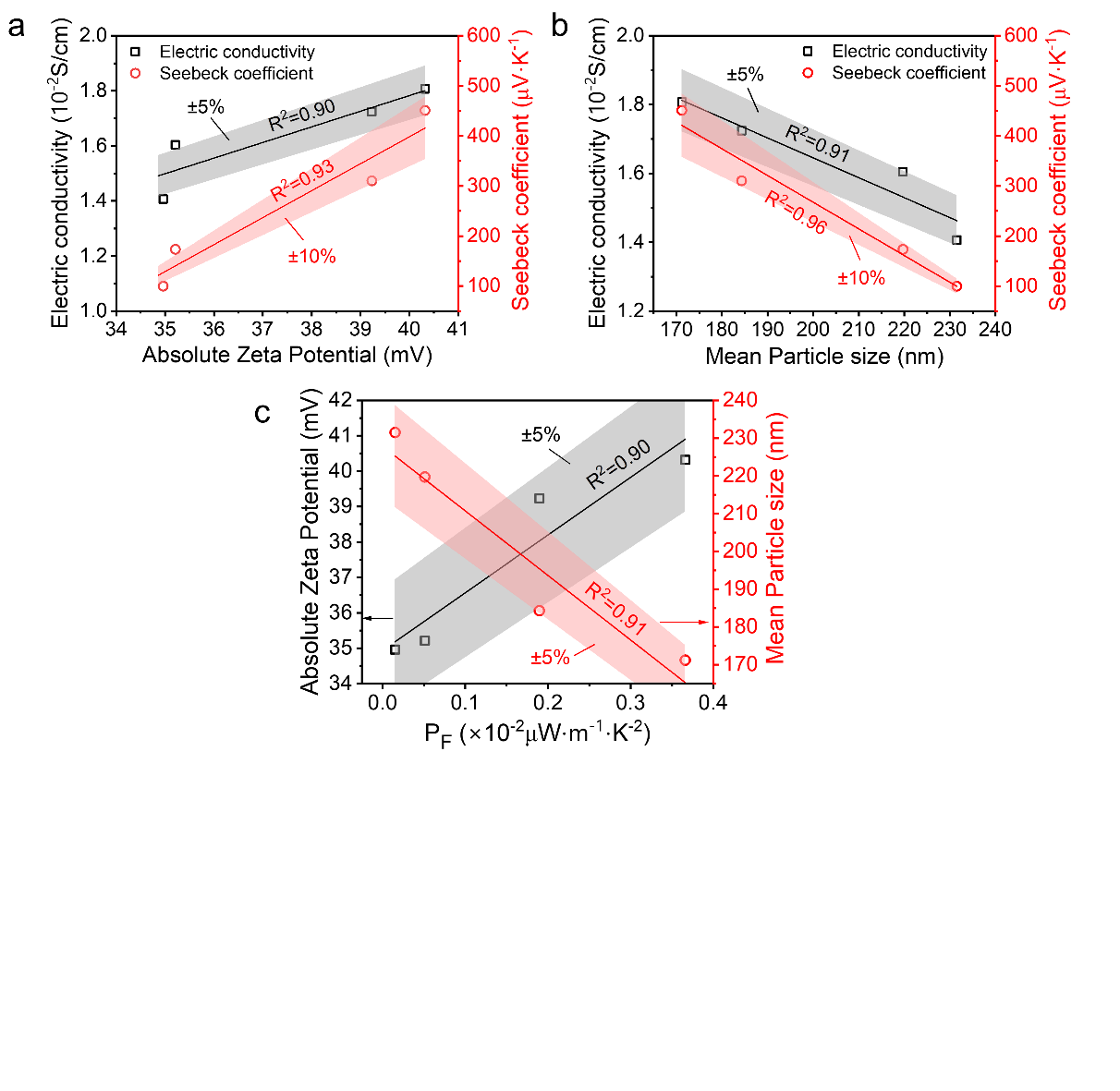


**Figure S20** The relationship between dispersibility and thermoelectric performance.

**Figure S21** Comparison of ZT value of multi-walled CNT cement-based materials.


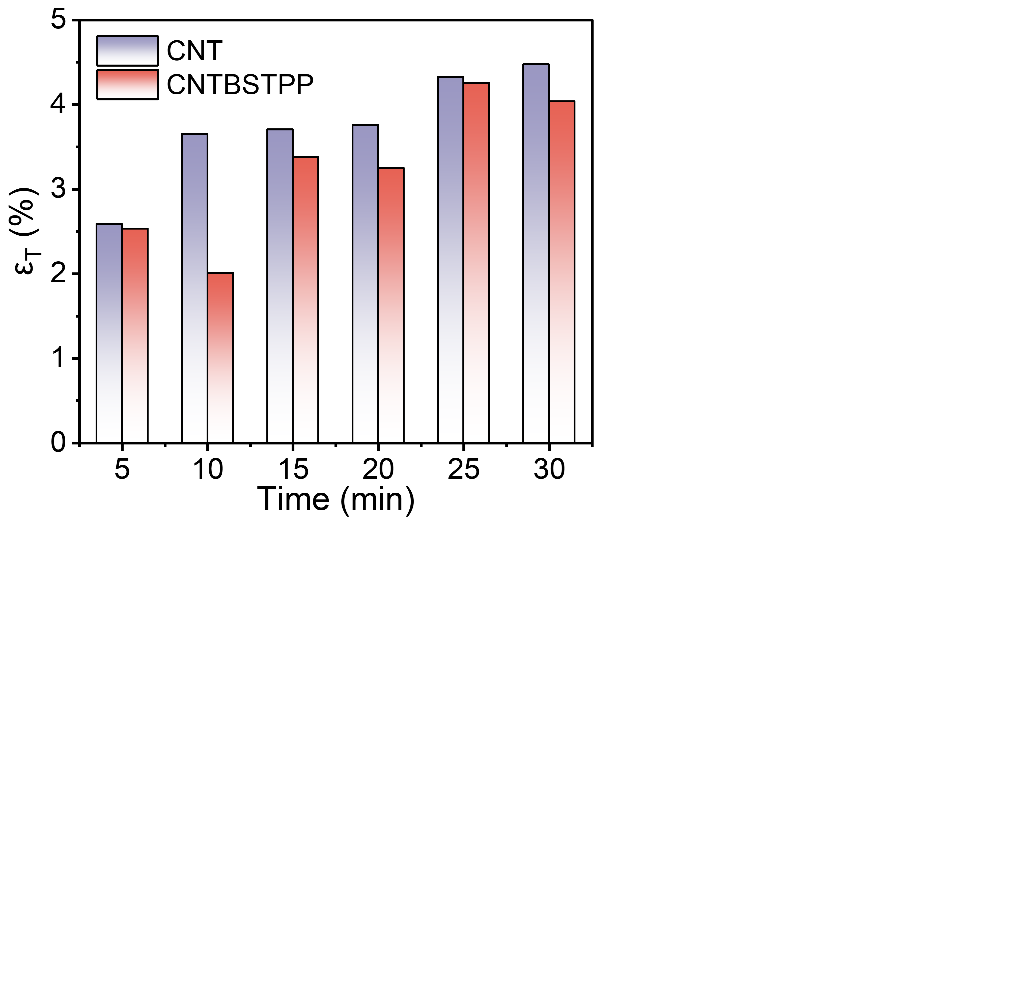


**Figure S22** Temperature difference distribution error ($\varepsilon_{T}$) of CNT and CNTBSTPP surfaces.

**
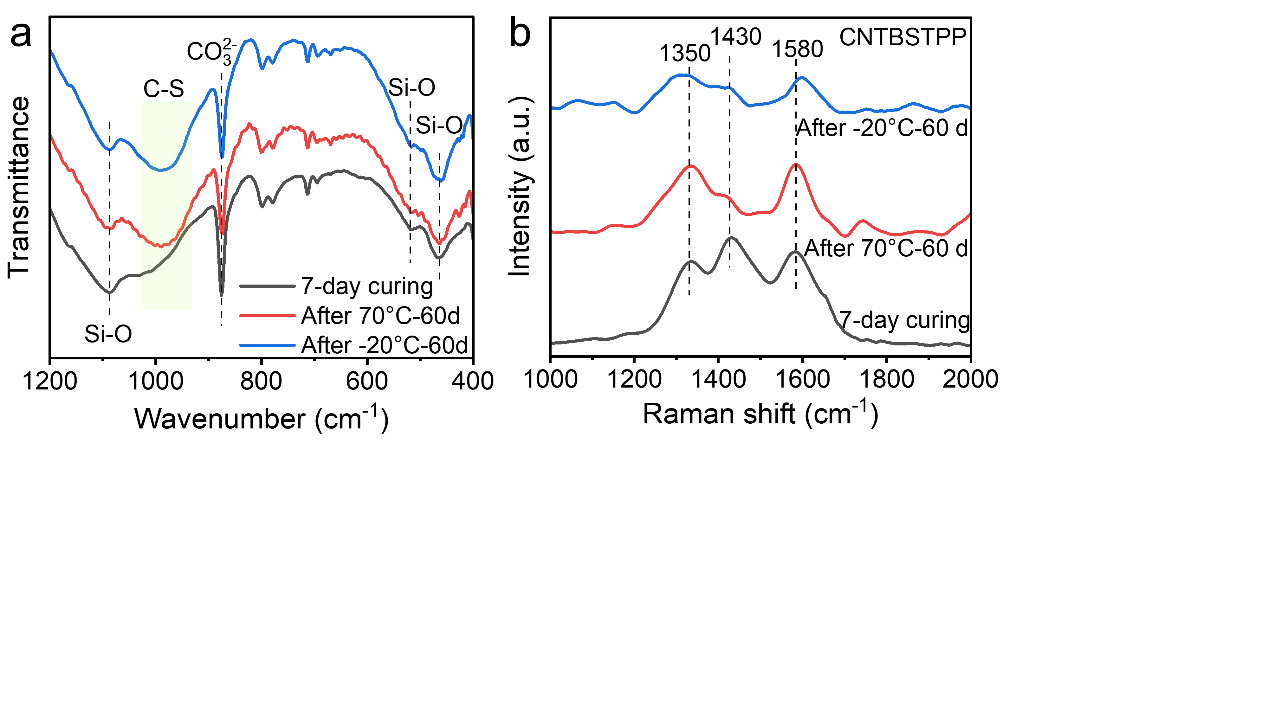
**

**Figure S23** (a) FTIR spectra and (b) Raman spectra after 60-day of high/low temperature.


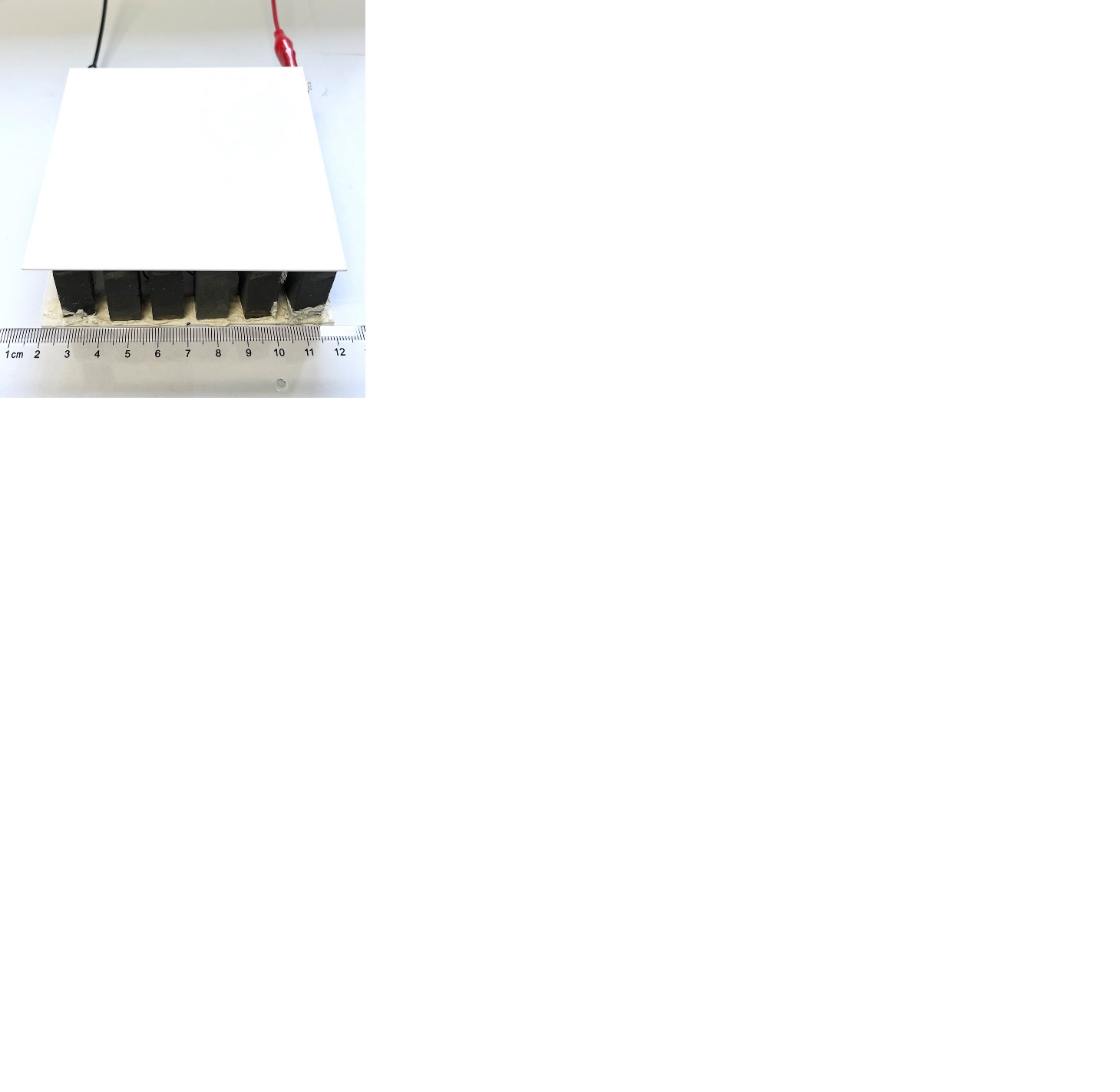


**Figure S24** The front view of TEG.


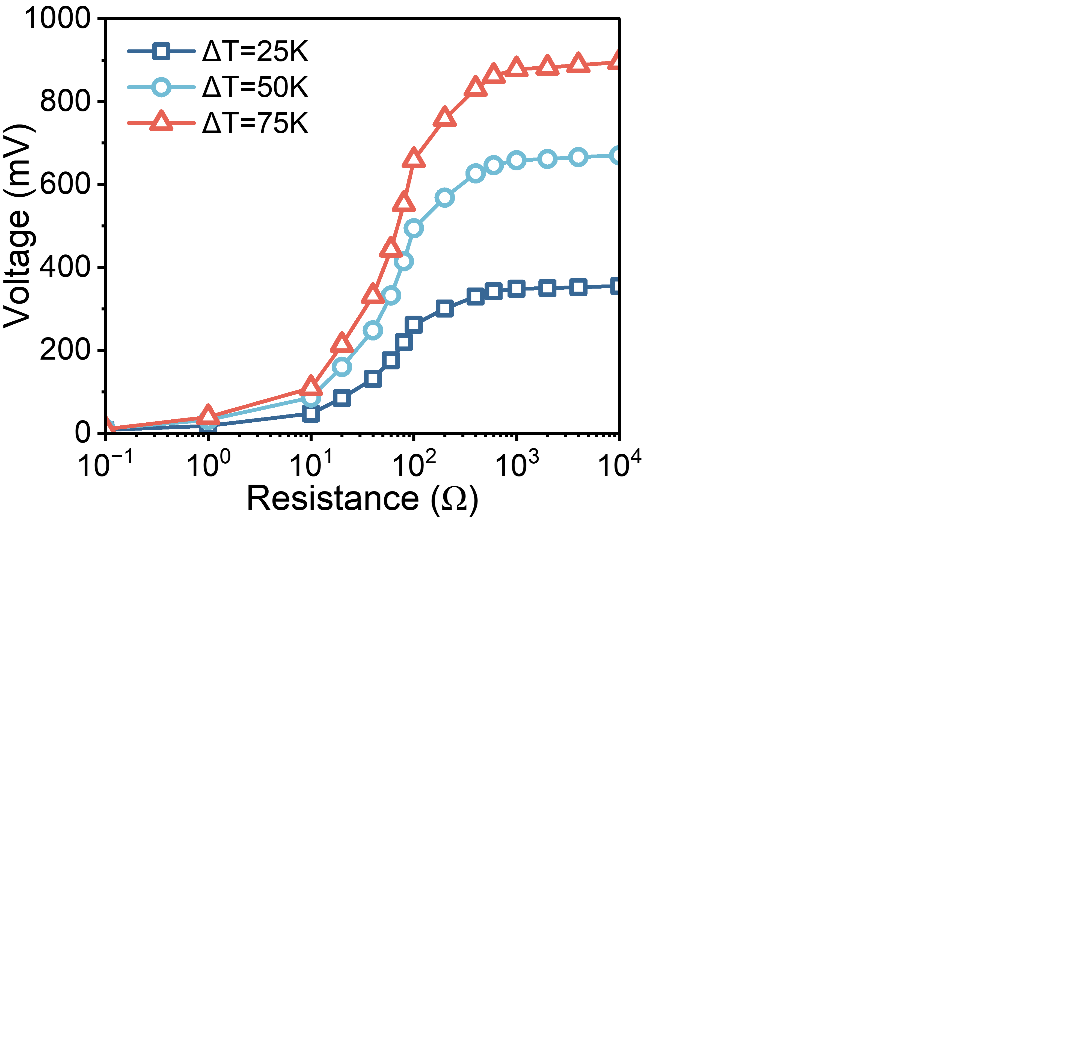


**Figure S25** Output voltage (*V*) -Load resistance (*R*_L_) of TEG.


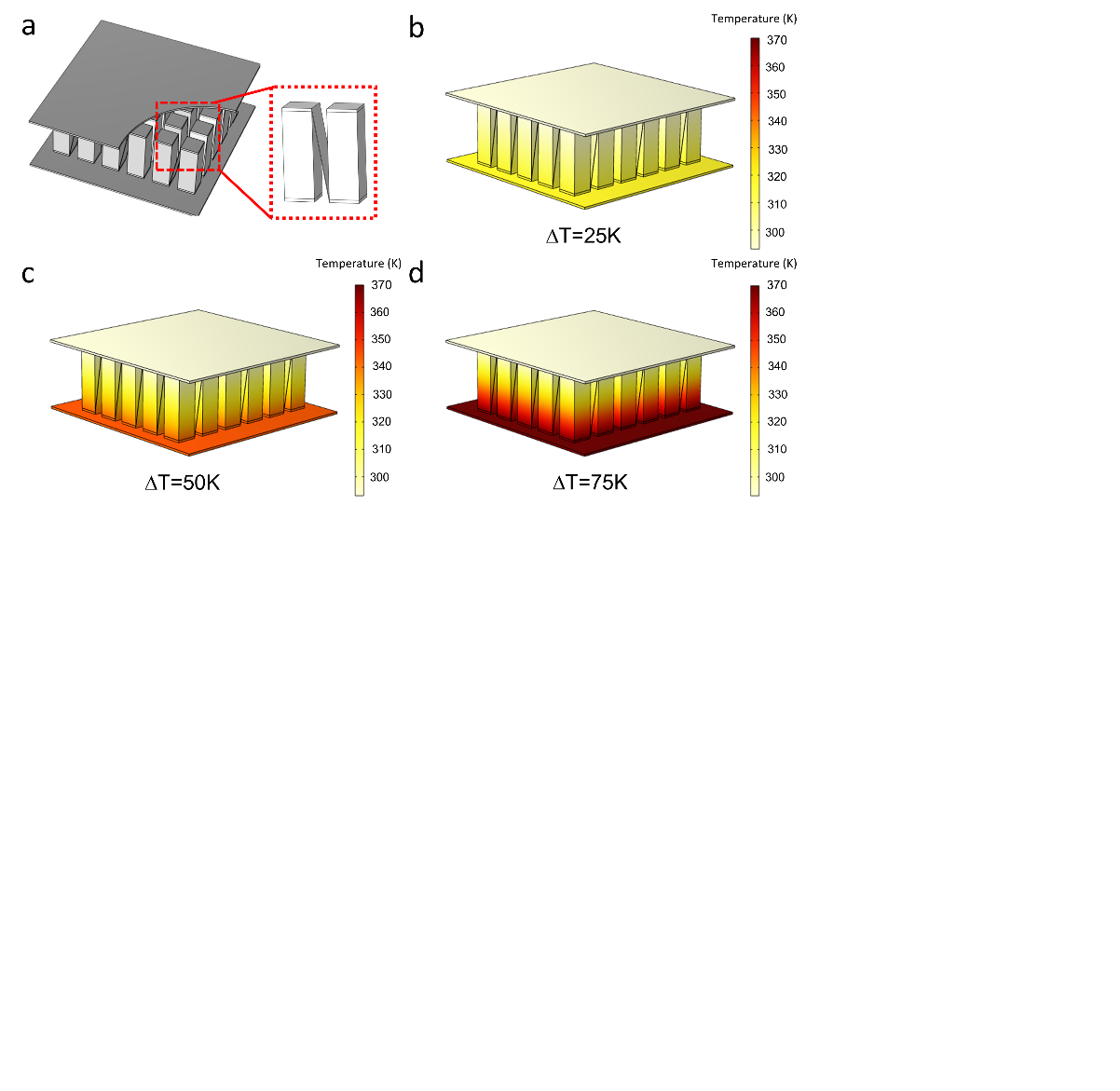


**Figure S26** (a) COMSOL multi-physics simulation diagram. TEG heat transfer simulation results: (b) ∆T=25K. (c) ∆T=50K. (d) ∆T=75K.


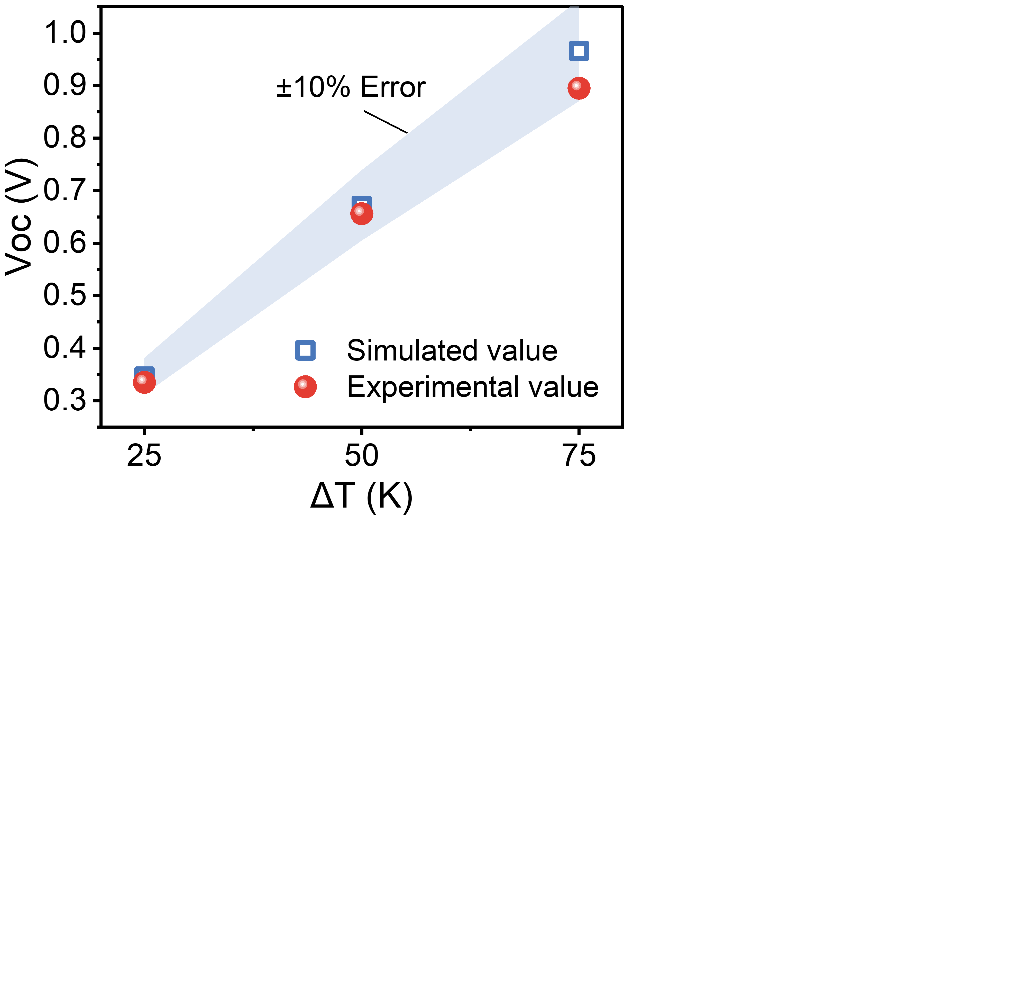


**Figure S27** The relationship between output voltage and simulated potential difference.

**
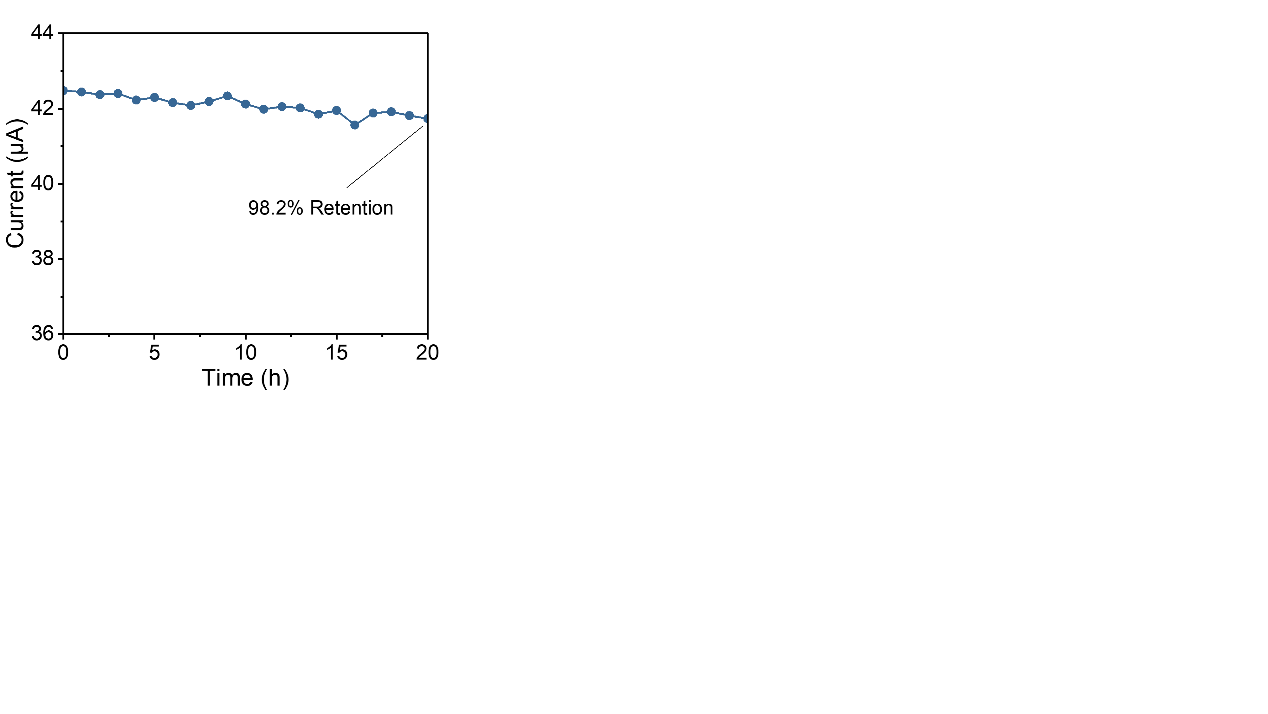
**

**Figure S28** The output current of TEG after continuous operation for 20 hours.


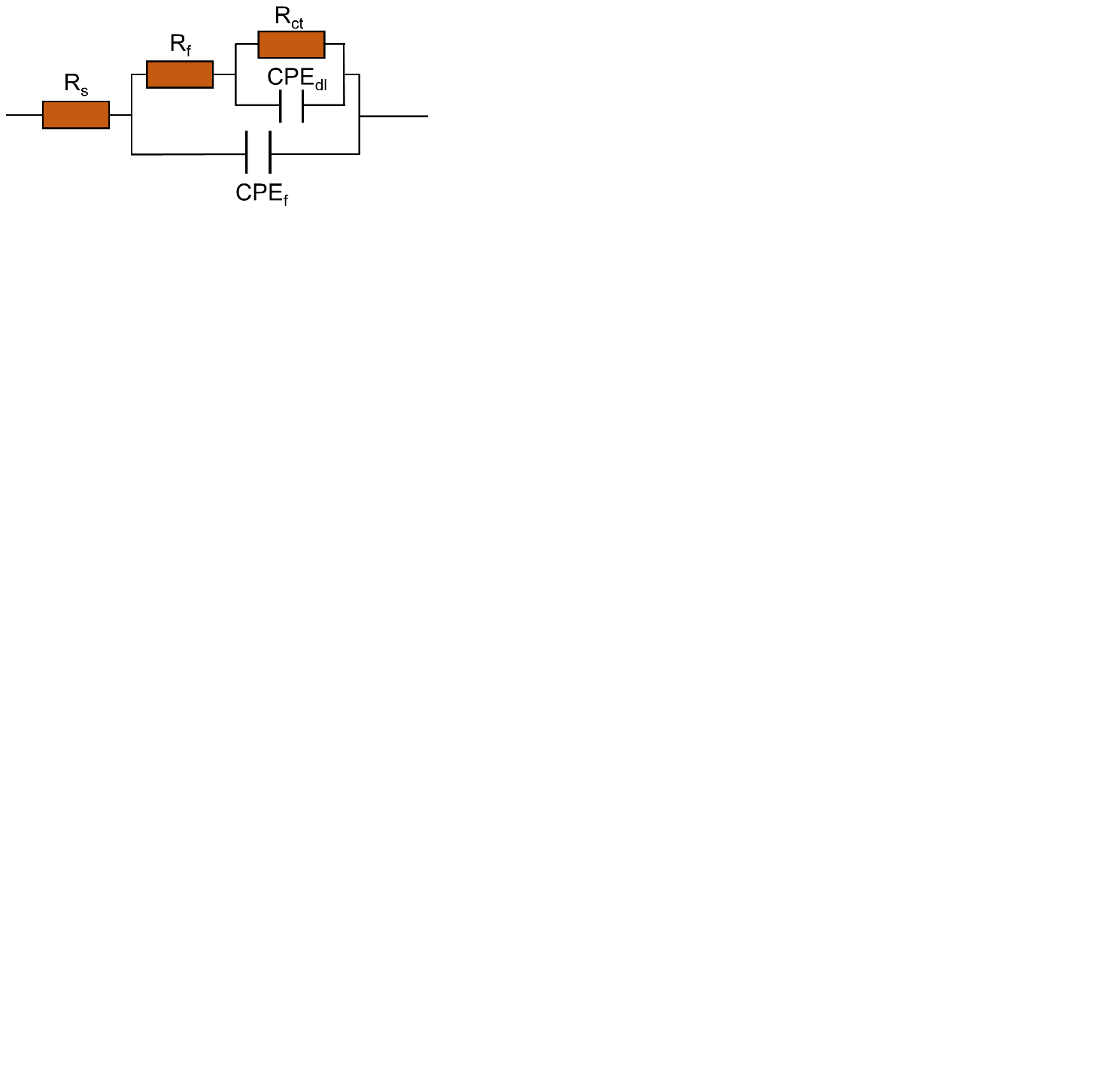


**Figure S29** EIS equivalent circuit diagram for cathodic protection.


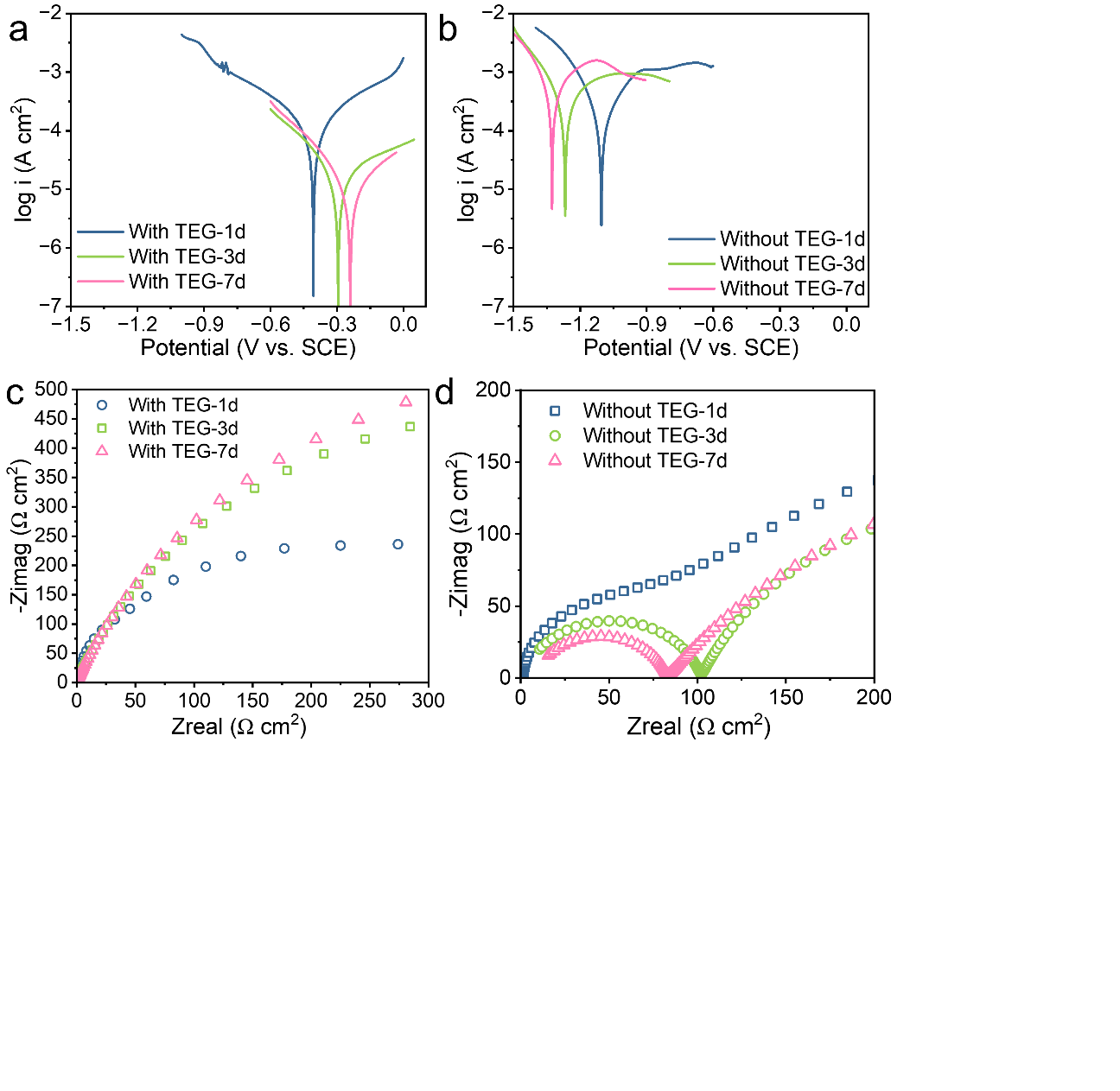


**Figure S30** (a), (b) Tafel and (c), (d) EIS results of A3 steel for 1d, 3d and 7d with and without TEG.


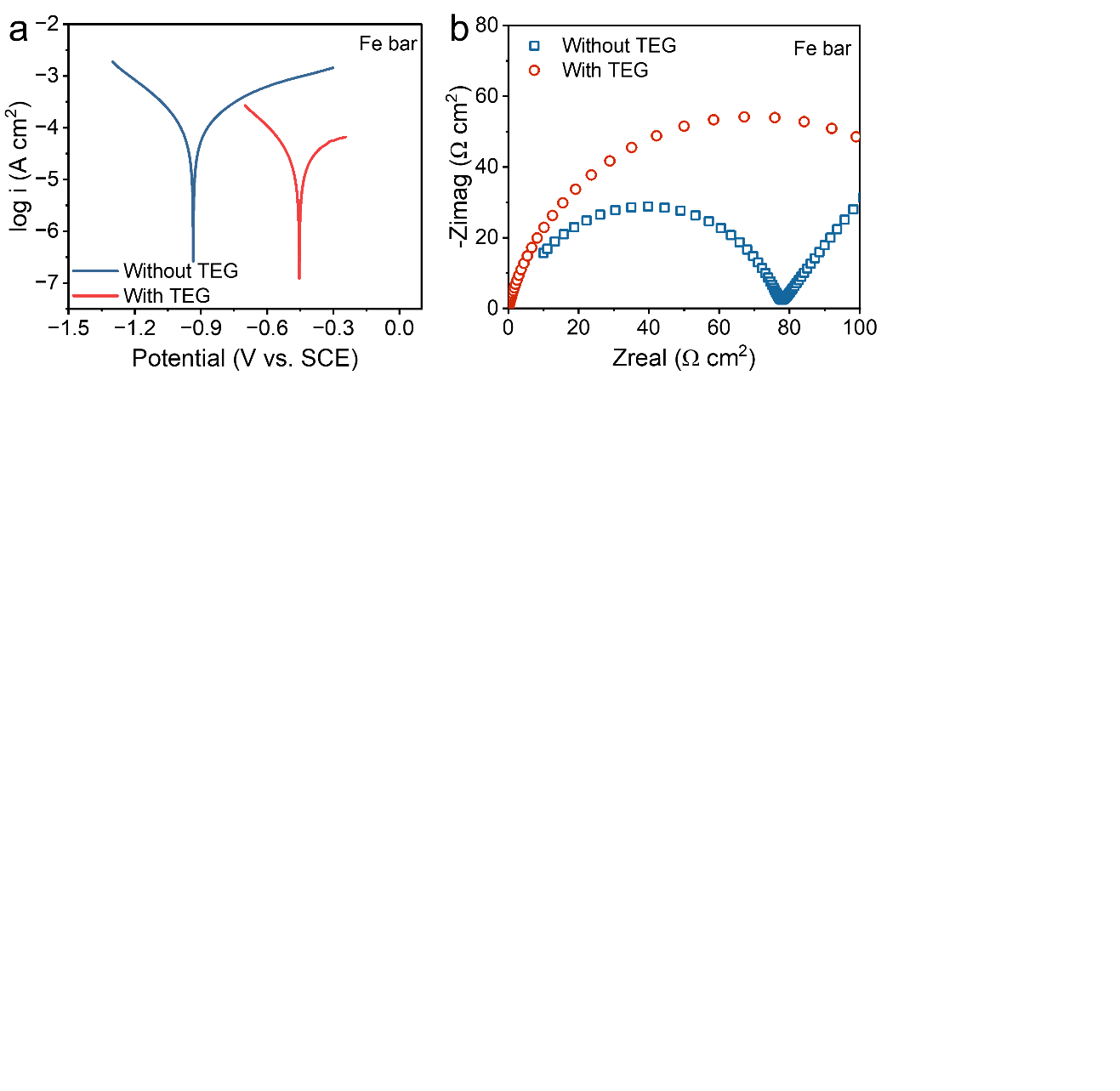


**Figure S31** (a) EIS and (b)Tafel results of Fe bar with and without TEG.


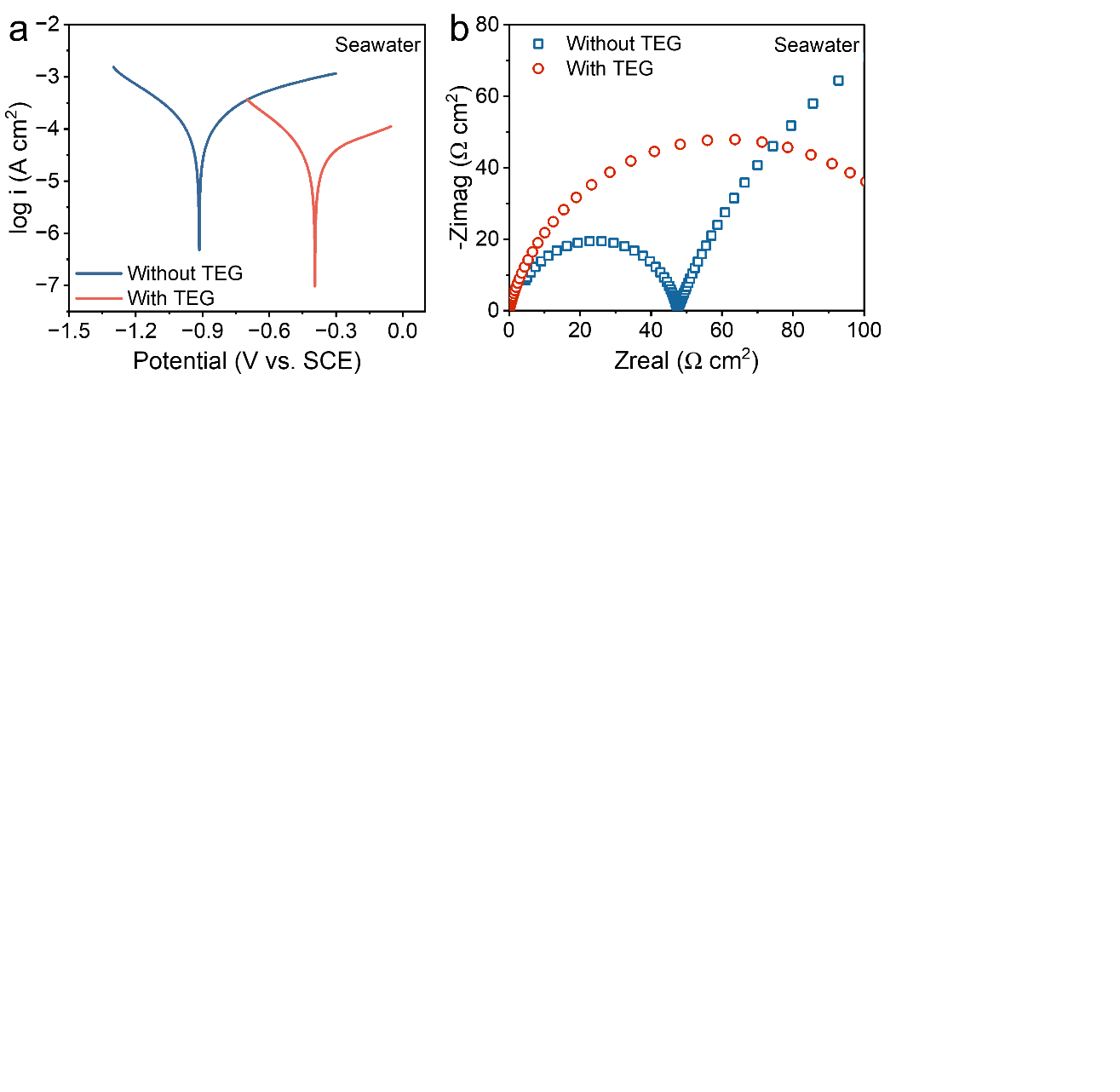


**Figure S32** (a) EIS and (b) Tafel results of A3 steel in seawater with and without TEG.

**Table S1** The area proportion of C 1s peak in XPS spectra (%).

| Samples | C-C | C-S/C-O | C-O-C | C-O=C | C=O |
| --- | --- | --- | --- | --- | --- |
| CNT | 21.73 | 22.17 | 16.36 | 33.55 | 6.20 |
| CNTPP | 22.74 | 30.23 | 12.27 | 30.92 | 3.82 |
| CNTBST | 22.10 | 25.59 | 12.95 | 34.74 | 4.62 |
| CNTBSTPP | 19.25 | 31.71 | 11.70 | 33.90 | 3.44 |

**Table S2** The area proportion of O 1s peak in XPS spectra (%).

| Samples | C-O | C=O |
| --- | --- | --- |
|  |  |  |
| CNT | 88.55 | 11.45 |
| CNTPP | 94.15 | 5.85 |
| CNTBST | 95.58 | 4.42 |
| CNTBSTPP | 95.19 | 4.81 |

**TableS3** Factors and level design.

| Factor |  | Level |  |
| --- | --- | --- | --- |
|  | 1 | 2 | 3 |
| A (CNT) | 0.10 wt% | 0.15 wt% | 0.20 wt% |
| B (BST) | 0.50 wt% | 1.00 wt% | 1.50 wt% |
| C (PEDOT: PSS) | 1.0 vol% | 2.00 vol% | 4.00 vol% |

**Table S4** Orthogonal test and results.

| Group | A  (CNT, wt%) | B  (BST, wt%) | C  (PEDOT: PSS, vol%) | σ  (S m^-1^) | S  (µV K^-1^) | Compressive strength  (MPa) |
| --- | --- | --- | --- | --- | --- | --- |
| 1 | 0.10 | 0.50 | 1 | 1.40 | 426.22 | 46.14 |
| 2 | 0.10 | 1.00 | 2 | 1.31 | 441.51 | 45.44 |
| 3 | 0.10 | 1.50 | 4 | 1.16 | 447.01 | 43.99 |
| 4 | 0.15 | 0.50 | 2 | 1.43 | 430.95 | 47.21 |
| 5 | 0.15 | 1.00 | 4 | 1.37 | 437.03 | 46.10 |
| 6 | 0.15 | 1.50 | 1 | 1.59 | 453.72 | 45.77 |
| 7 | 0.20 | 0.50 | 4 | 1.51 | 430.73 | 45.73 |
| 8 | 0.20 | 1.00 | 1 | 1.81 | 450.42 | 46.32 |
| 9 | 0.20 | 1.50 | 2 | 1.63 | 462.71 | 44.70 |

**Table S5** Range analysis of test results.

| Index | σ (S cm^-1^) | | | S (µV K^-1^) | | | Compressive strength (MPa) | | |
| --- | --- | --- | --- | --- | --- | --- | --- | --- | --- |
|  | A | B | C | A | B | C | A | B | C |
| $\overline{K_{1j}}$ | 1.29 | 1.44 | 1.60 | 438.24 | 429.30 | 443.45 | 45.19 | 46.36 | 46.08 |
| $\overline{K_{2j}}$ | 1.46 | 1.50 | 1.46 | 440.57 | 442.99 | 445.60 | 46.36 | 45.95 | 45.78 |
| $\overline{K_{3j}}$ | 1.65 | 1.46 | 1.37 | 447.95 | 454.48 | 438.26 | 45.58 | 44.82 | 45.28 |
| *R_j_* | 0.36 | 0.06 | 0.23 | 9.71 | 25.18 | 6.80 | 1.17 | 1.54 | 0.80 |
| Level | 3 2 1 | 2 3 1 | 1 2 3 | 3 2 1 | 3 2 1 | 2 1 3 | 2 3 1 | 1 2 3 | 1 2 3 |
| Factor | A: C: B | | | B: A: C | | | B: A: C | | |

**Table S6** Electrochemical impedance parameters for CNT/PP/BST cement-based materials.

| Samples | R_s_ /Ω cm^2^ | CPE_dl_ | | R_ct_/kΩ cm^2^ | Zw |
| --- | --- | --- | --- | --- | --- |
|  |  | Y_0_ | n |  |  |
| CNT | 8.694×10^1^ | 2.609×10^-10^ | 0.618 | 10.43 | 4.703×10^-5^ |
| CNTPP | 1.687×10^2^ | 2.213×10^-8^ | 0.690 | 7.049 | 9.668×10^-5^ |
| CNTBST | 1.763×10^2^ | 2.594×10^-8^ | 0.649 | 9.360 | 1.817×10^-5^ |
| CNTBSTPP | 1.535×10^2^ | 2.721×10^-8^ | 0.628 | 4.568 | 7.716×10^-5^ |

**Table S7** Comparison of thermoelectric properties of multi-walled CNT cement-based materials.

| Material | σ  (S cm^-1^) | S  (μV K^-1^) | κ  (W m^−1^ K^−1^) | PF  (μW m^−1^ K^−2^) | ZT | Reference |
| --- | --- | --- | --- | --- | --- | --- |
| 0.125%CNT | 3.04× 10^-3^ | 14.45 | 1.95 | 6.35× 10^-7^ | 9.78× 10^-11^ | [1] |
| 0.2%CNT | 5.56× 10^-3^ | 15.41 | 1.91 | 1.32× 10^-6^ | 2.07× 10^-10^ | [1] |
| 0.25%CNT | 2.52 | 13.67 | 1.89 | 4.71 × 10^-6^ | 7.48 × 10^-10^ | [1] |
| 15%CNT | 0.82 | 57.98 | 0.734- 0.947 | 0.25 | 9.33 × 10^-5^ | [2] |
| 10%CNT+Li_2_CO_3_ | 0.07 | 73.40 | 0.839 | 0.039 | 1.75 × 10^-5^ | [3] |
| 5%HCl-CNT | 0.22 | 66 | - | 0.12 | 0.38× 10^-4^ | [4] |
| 1% CNT | 2.21 | 59 ± 2.2 | - | 7.63× 10^-3^ | - | [5] |
| 0.3%p-CNT | 5.32× 10^-2^ | 86.7 |  | 4× 10^-4^ |  | [6] |
| 0.3%n-CNT | 5.34× 10^-2^ | -14.9 |  | 1× 10^-5^ |  | [6] |
| 0.4%CF+0.5%  CNT | 1.41× 10^-3^ | 21.70 | - | 6.63× 10^-5^ | - | [7] |
| 0.5%SrTiO3+1.0%CNT | 1.1× 10^-4^ | 1100 | - | 0.65 × 10^-6^ | - | [8] |
| 1.0%SrTiO3+1.0%CNT | 1.0× 10^-4^ | 2000 | - | 1.61 × 10^-6^ | - | [8] |
| 0.2%CNT+1.0%BST+1.0%PP | 1.81× 10^-2^ | 450 | 0.458 | 4.18× 10^-5^ | 2.39×10⁻^4^ | This work |

**Table S8** Fitting data of polarization curves of steel rebars.

| Samples | Corrosion Voltage/V | Corrosion Current/µA cm^-2^ | Corrosion rate/mm a^-1^ | Linear polarization resistance/Ω cm^2^ |
| --- | --- | --- | --- | --- |
| Without TEG | -1.104 | 1.63 | 7.42 | 197 |
| With TEG | -0.409 | 0.86 | 3.93 | 507 |

**Table S9** Electrochemical impedance parameters for A3 steel with and without TEG.

| Samples | R_s_ /Ω cm^2^ | CPE_f_ | | R_f_ /Ω cm^2^ | CPE_dl_ | | R_ct_/Ω cm^2^ |
| --- | --- | --- | --- | --- | --- | --- | --- |
|  |  | Y_0_ | n |  | Y_0_ | n |  |
| Without TEG | 1.81 | 1.59×10^-3^ | 1.0 | 476.1 | 2.13×10^-3^ | 1.0 | 8.09 |
| With TEG | 1.46 | 7.18×10^-3^ | 1.0 | 95.96 | 2.99×10^-2^ | 0.699 | 4.89×10^2^ |

**Table S10** Fitting data of Tafel curves with and without TEG.

| Samples | E_corr_ /V | i_corr_ /µA cm^-2^ | β_c_/mV dec^-1^ | β_a_/mV dec^-1^ |
| --- | --- | --- | --- | --- |
| Without TEG-1d | -1.104 | 1.63 | -5361 | 4502 |
| Without TEG-3d | -1.267 | 2.05 | -5316 | 3907 |
| Without TEG-7d | -1.326 | 2.21 | -5068 | 4479 |
| With TEG-1d | -0.409 | 0.86 | -5416 | 4523 |
| With TEG-3d | -0.294 | 0.68 | -6007 | 4019 |
| With TEG-7d | -0.241 | 0.56 | -6260 | 4639 |
| Without TEG-Fe bar | -0.936 | 1.82 | -5287 | 4276 |
| With TEG-Fe bar | -0.454 | 0.96 | -5438 | 4456 |
| Without TEG-seawater | -0.915 | 1.65 | -5543 | 4557 |
| With TEG-seawater | -0.395 | 0.89 | -5900 | 3800 |

**Table S11** Electrochemical impedance parameters with and without TEG.

| Samples | R_s_/Ω cm^2^ | CPE_f_ | | R_f_ /Ω cm^2^ | CPE_dl_ | | R_ct_/Ω cm^2^ |
| --- | --- | --- | --- | --- | --- | --- | --- |
|  |  | Y_0_ | n |  | Y_0_ | n |  |
| Without TEG-1d | 1.81 | 1.59×10^-3^ | 1.0 | 476.10 | 2.13×10^-3^ | 1.0 | 8.09 |
| Without TEG-3d | 2.09 | 2.08×10^-3^ | 0.91 | 7.34 | 5.01×10^-4^ | 1.0 | 5.99 |
| Without TEG-7d | 2.04 | 4.99×10^-3^ | 0.83 | 7.59 | 1.13×10^-4^ | 0.62 | 5.47 |
| With TEG-1d | 1.46 | 7.18×10^-3^ | 1.0 | 95.96 | 2.99×10^-4^ | 0.69 | 489.2 |
| With TEG-3d | 1.94 | 5.45×10^-3^ | 0.97 | 111.8 | 1.85×10^-4^ | 0.78 | 549.14 |
| With TEG-7d | 1.88 | 2.89×10^-3^ | 0.80 | 101.1 | 2.68×10^-4^ | 0.80 | 553.4 |
| Without TEG-Fe bar | 1.85 | 4.38×10^-3^ | 0.85 | 121.4 | 1.87×10^-4^ | 1.0 | 2.41 |
| With TEG-Fe bar | 1.09 | 1.99×10^-3^ | 1.0 | 125.8 | 4.81×10^-4^ | 0.89 | 308.8 |
| Without TEG-seawater | 1.46 | 4.95×10^-3^ | 0.85 | 136.9 | 1.72×10^-4^ | 1.0 | 2.6 |
| With TEG-seawater | 1.88 | 1.27×10^-3^ | 0.87 | 165.8 | 2.13×10^-4^ | 0.78 | 395.2 |

**Table S12** Chemical composition of cement (%).

| CaO | SiO_2_ | Al_2_O_3_ | Fe_2_O_3_ | SO_3_ | MgO | K_2_O | Rest |
| --- | --- | --- | --- | --- | --- | --- | --- |
| 64.58 | 23.55 | 5.16 | 2.76 | 1.83 | 1.32 | 0.36 | 0.44 |

**Table S13** Physical properties of MWCNTs.

| Inner  diameter  /nm | Outer diameter  /nm | Length  /µm | Specific surface area  /m^2^·g^-1^ | Density  /g·cm^-3^ | Resistivity  /µΩ·m |
| --- | --- | --- | --- | --- | --- |
| 3-5 | 8-15 | 3-12 | 233 | 0.15 | 1412 |

# References

1. Kyungwho C, Daeyeon K, Wonseok C, et al. Nanostructured thermoelectric composites for efficient energy harvesting in infrastructure construction applications. Cement and Concrete Composites,2022, 104452.
2. Wei J, Fan Y, Zhao L, et al. Thermoelectric properties of carbon nanotube reinforced cement-based composites fabricated by compression shear. Ceramics International,2018,44(6):5829-5833.
3. Jian W, Mengjie Z, Yuan W, et al. Synergistic optimization of thermoelectric performance in cementitious composites by lithium carbonate and carbon nanotubes. International Journal of Energy Research,2020,45(2):2460-2473.
4. Jian W, Zhuang M, Yuan W, et al. Boosting power factor of thermoelectric cementitious composites by a unique CNT pretreatment process with low carbon content. Energy & Buildings,2022,254.
5. Tzounis L, Liebscher M, Fuge R, et al. P- and n-type thermoelectric cement composites with CVD grown p- and n-doped carbon nanotubes: Demonstration of a structural thermoelectric generator. Energy & Buildings,2019,191151-163.
6. Soonho Kim, Rongzhen Piao, Seung Kyun Lee, et al. Thermoelectric cement-based composites containing carbon nanotubes (CNTs): Effects of water-to-cement ratio and CNT dosage. Case Studies in Construction Materials, 2024(21), e03861.
7. Zuo J, Yao W, Wu K. Seebeck Effect and Mechanical Properties of Carbon Nanotube-Carbon Fiber/Cement Nanocomposites. Fullerenes, Nanotubes and Carbon Nanostructures,2015,23(5):383-391.
8. Didi H, Fengyu S, Yanmin W, et al. Carrier type control in cement-based thermoelectric composites with carbon nanotube and SrTiO3 nanoparticles. Materials Today Communications,2023,37.
9. H. J. Goldsmid, J. W. Sharp, J. Electron. Mater. **1999**, 28, 869.
10. H.-S. Kim, Z. M. Gibbs, Y. Tang, H. Wang, G. J. Snyder, APL Mater. **2015**, 3, 041506.
